# Neuronal spreading and plaque induction of intracellular Aβ and its disruption of Aβ homeostasis

**DOI:** 10.1007/s00401-021-02345-9

**Published:** 2021-07-16

**Authors:** Tomas T. Roos, Megg G. Garcia, Isak Martinsson, Rana Mabrouk, Bodil Israelsson, Tomas Deierborg, Asgeir Kobro-Flatmoen, Heikki Tanila, Gunnar K. Gouras

**Affiliations:** 1 Experimental Dementia Research Unit, Department of Experimental Medical Science, Lund University, Lund, Sweden; 2 Experimental Neuroinflammation Laboratory, Department of Experimental Medical Science, Lund University, Lund, Sweden; 3 A. I. Virtanen Institute, University of Eastern Finland, Kuopio, Finland; 4 Kavli Institute for Systems Neuroscience, Trondheim, Norway

**Keywords:** Alzheimer’s disease, Amyloid, Prion-like, Hippocampus, Entorhinal cortex, Interneurons

## Abstract

The amyloid-beta peptide (Aβ) is thought to have prion-like properties promoting its spread throughout the brain in Alzheimer’s disease (AD). However, the cellular mechanism(s) of this spread remains unclear. Here, we show an important role of intracellular Aβ in its prion-like spread. We demonstrate that an intracellular source of Aβ can induce amyloid plaques in vivo via hippocampal injection. We show that hippocampal injection of mouse AD brain homogenate not only induces plaques, but also damages interneurons and affects intracellular Aβ levels in synaptically connected brain areas, paralleling cellular changes seen in AD. Furthermore, in a primary neuron AD model, exposure of picomolar amounts of brain-derived Aβ leads to an apparent redistribution of Aβ from soma to processes and dystrophic neurites. We also observe that such neuritic dystrophies associate with plaque formation in AD-transgenic mice. Finally, using cellular models, we propose a mechanism for how intracellular accumulation of Aβ disturbs homeostatic control of Aβ levels and can contribute to the up to 10,000-fold increase of Aβ in the AD brain. Our data indicate an essential role for intracellular prion-like Aβ and its synaptic spread in the pathogenesis of AD.

**Supplementary Information:**

The online version contains supplementary material available at 10.1007/s00401-021-02345-9.

## Introduction

Alzheimer’s disease (AD) is neuropathologically characterized by large extracellular plaques of amyloid-beta peptide (Aβ) and intracellular tangles of microtubule-associated protein tau. Genetic and clinical evidence implicates Aβ as playing a key role in initiating AD. All known dominantly inherited forms of AD are due to genetic mutations that either lead to production of more Aβ or more aggregation-prone forms of Aβ [37]. Furthermore, a genetic polymorphism that results in less Aβ protects against AD [13]. Moreover, the first detectable biomarker change in AD is a decline of cerebrospinal fluid (CSF) Aβ42, which can be observed up to 2 decades before the onset of dementia [2]. Yet, we still do not know how Aβ starts to aggregate and spread throughout the brain.

One possible explanation that has gained traction in recent years is that Aβ has prion-like properties [40]. This hypothesis proposes that misfolded Aβ spreads via templated seeding, meaning that once a prion-like seed of Aβ is formed, it could propagate its misfolded structure throughout the brain. The formation of prion-like Aβ would then be essential to the early pathogenesis of AD. Supporting this idea, studies have shown that the formation of Aβ plaques can be accelerated, or seeded, in several mouse and rat models of AD by intracerebral injection of AD brain homogenate [15, 19, 21]. Still, only some forms of Aβ aggregates appear capable of prion-like seeding, and their identities are not precisely known. For example, small amounts of soluble brain-derived Aβ can induce seeding [7], while synthetic Aβ requires large amounts and extended fibrillization [32] and, interestingly, Aβ-containing CSF from AD patients does not seed at all [7]. Unilateral injection of AD brain homogenate into the hippocampus of APP23 AD-transgenic mice resulted, 30 days later, in almost tenfold higher Aβ levels in the injected side compared to the non-injected side despite no indication of plaque induction by that time [46]. This is consistent with a pre-plaque aggregation phase by the introduction of prion-like Aβ. It also indicates markedly increased Aβ production and/or decreased degradation before the onset of plaques. Previous studies have examined the anatomical spread of induced Aβ [39, 47], but they have focused on the appearance of extracellular plaques and not on the intracellular localization of early Aβ peptide aggregation. Thus, more studies are needed to elucidate the mechanisms behind the seeding of Aβ.

In this study, we show that a cellular source of Aβ can induce plaques in vivo*,* providing a further clue as to what types of Aβ are capable of prion-like seeding. Furthermore, we study the anatomy of early AD brain homogenate-induced plaques and the progression of amyloid induction in the brain at various time points. We also observe neuronal damage in hippocampus and changes in intracellular Aβ and dystrophic neurites in anatomically connected regions. Finally, we examine potential mechanisms of how accumulation of intracellular Aβ induces its own production that may explain the massive increase in Aβ seen in the brains of both AD-model transgenic mice [46] and human AD patients [28]. Taken together, our data point toward an important cellular phase in the prion-like propagation of Aβ.

## Materials and methods

### Animals

All animal experiments were ethically approved by the Malmö/Lund Ethics Committee on Animal Testing (dnr. M46-16 and M12561-20) and compliant with the ethical guidelines on animal experiments in Sweden (SJVFS 2019:9, saknr L 150) and European directive 2010/63/EU. For primary neurons and brain homogenate, we used amyloid precursor protein (APP)/presenilin 1 (PSEN1) AD mutant mice (Jackson laboratory, B6C3-Tg [APPswe,PSEN1dE9] 85Dbo/Mmjax), C57Bl/6 mice (wild-type), and APP KO mice (Jackson Laboratory, B6.129S7-App^tm1Dbo^/J). For the injection experiments, we used 5xFAD mice (Jackson Laboratory, B6SJL-Tg [APPSwFlLon,PSEN1*M146L*L286V] 6799Vas/Mmjax) and B6SJL (WT) of both sexes. A subset of 5xFAD mice were cross-bred with Thy1-GFPM mice (Jackson Laboratory, Tg[Thy1-EGFP]MJrs/J), and female offspring were used for the injection of viral vectors into the entorhinal cortex (ErC).

### Preparation of brain homogenate

The forebrains of 21-month-old APP/PSEN1 or wild-type (WT) mice were collected, immediately frozen on dry ice, and stored at − 80 °C. The forebrains were homogenized in 10% weight/volume sterile phosphate-buffered saline (PBS), sonicated 3 times for 5 s at 80% amplitude with a Branson SLPe model 4C15 sonifier, and then centrifuged at 3000×*g* for 5 min at 4 °C. The resulting supernatant was sonicated three times for 20 s each at 80% amplitude as described in Langer et al. [19]. The supernatant was then aliquoted and kept at − 80 °C until used for intracerebral injections or centrifuged at 100,000×*g* for 1 h at 4 °C to produce ultracentrifuged brain homogenate used in the primary neuron experiments.

### Intracerebral injections

Intracerebral injections into hippocampus were performed as described in Meyer-Luehmann et al. [21]. Briefly, mice were anesthetized with isoflurane, and their heads were shaved and sterilized with 70% ethanol and then placed in a stereotactic frame. An incision was made to expose the skull and a small hole was drilled at the following coordinates from Bregma, for hippocampal injections: AP − 2.5, ML 2.0, DV − 1.8; entorhinal injections at AP − 2.3, ML 2.5, DV − 2.0 (from dura). For hippocampal injections, 5 µl of brain homogenate, cell lysate, or a mixture of 4.2 µl brain homogenate and 0.8 µl India ink was injected at a speed of 1.25 µl/min via a Hamilton syringe. For entorhinal injections, the ErC of 5xFAD × Thy1-GFPM female mice was injected with 1 µl of AAV-mCherry at the age of 2 months. The needle was then kept in place for 2 min to allow for diffusion of material and then slowly withdrawn. The skin incision was then sutured, and the mice were monitored until recovery from anesthesia. Weight and behavior were monitored after surgery.

### Primary neuron cultures

Primary neuronal cultures were performed according to ethical guidelines set by the ethics committee for the use of laboratory animals in Lund University (M5983-19). Primary cortico-hippocampal cultures were harvested from APP/PSEN1((APPswe, PSEN1dE9)85Dbo/Mmjax) transgenic mice (Jackson Labs, Maine, USA) at embryonic day 16. Pregnant mice were deeply anaesthetized with isoflurane (MSD Animal Health, Sweden) and sacrificed. Embryos were quickly removed and biopsied for genotyping to identify which embryos are wild type and which carry the transgene. Brains were dissected under constant cooling with chilled (≈ 4 °C) Hank’s balanced salt solution (HBSS, Thermo Fisher Scientific, Sweden) supplemented with 0.45% glucose. Cortices and hippocampi were dissected and then incubated with 0.05% trypsin (Thermo Fisher Scientific, Sweden) for 15 min at 37 °C. After incubation with trypsin, samples were rinsed two times with HBSS 0.45% glucose buffer. Brain tissue was then triturated using glass pipettes in 10% fetal bovine serum (FBS) supplemented Dulbecco’s modified Eagle Media (DMEM, Thermo Fisher Scientific, Sweden). Neurons were plated onto Poly-d-Lysine (PDL, Sigma-Aldrich, Sweden) coated coverslips in 24-well plates for immunofluorescence (≈ 35,000 cells/well), or coated wells in 24-well plate for dot blot (≈ 70,000 cells/well). Neurons were plated in 10% FBS and 1% penicillin–streptomycin supplemented DMEM and allowed to attach for 3–5 h. After neurons attach culture media are exchanged for complete Neurobasal medium supplemented with B27, penicillin–streptomycin, and l-glutamine (Thermo Fisher Scientific, Sweden).

### N2a cells

N2a neuroblastoma cells were grown in media-containing 47% high-glucose DMEM, 47% Opti-MEM, 5% FBS, and 1% penicillin/streptomycin (Thermo Fisher Scientific for all) at 37 °C in a humid 5% CO_2_ incubator. For cell collection, the cells were washed twice on ice with ice-cold PBS and collected with a cell scraper. Cells were then pelleted at 10,600×*g* for 2 min at 4 °C, snap-frozen in liquid nitrogen, and stored at − 80 °C.

### Perfusion and tissue collection

Intracerebrally injected 5xFAD mice were transcardially perfused under deep anesthesia with 0.1 M, pH 7.4 ice-cold phosphate buffer (PB) followed by ice-cold 4% paraformaldehyde (PFA). Brains were dissected out and post-fixed overnight in 4% PFA. Brains were then incubated in a series of sucrose solutions, beginning with 15% and ending at 30%, and remained in each until they sank to the bottom. Brains were held in the last 30% sucrose solution until sectioned. For sectioning, brains were chilled with dry ice and coronally sectioned to a thickness of 30 µm using a sledge microtome. Sections were stored in cryopreserve (30% sucrose and 30% ethylene glycol in 0.1 M PB, pH 7.4) at − 20 °C. 5xFAD × Thy1-GFPM mice were perfused at 4 months of age with 0.9% saline followed by 4% PFA. Brains were left overnight in 30% sucrose and then kept in antifreeze at − 20 °C until sectioned into 35 µm-thick coronal sections.

### Antibodies

We used the following secondary antibodies: AF488 anti-mouse and anti-rabbit (Thermo Fisher), CY3 anti-rabbit (Jackson ImmunoResearch); mouse IgG HRP-conjugated antibody, and Rabbit IgG HRP-conjugated antibody (R&D Systems).

### Immunolabeling

All brain labeling except for those in Fig. 7e, f were performed with the following protocol: brain sections were washed 5 min × 3 in 0.1 M PBS under gentle agitation. To better stain, intraneuronal Aβ sections were treated with 88% formic acid 12% 0.1 M PBS for 8 min [4]. Sections were then washed in 0.1 M PBS with 0.25% Triton-X 5 min × 3. Sections were blocked in 0.1 M PBS 3% normal goat serum (NGS, Jackson Immuno) and 0.25% Triton-X for 1 h. Sections were then incubated overnight with primary antibody in 0.1 M PBS, 3% NGS, and 0.25% Triton-X under gentle agitation at 4 °C. Sections were washed for 10 min × 3 in 0.1 M PBS with 0.25% Triton-X. The sections were then incubated with appropriate secondary antibody for 1 h in 0.1 M PBS, 3% NGS, and 0.25% Triton-X under gentle agitation in the dark at room temperature. Sections were washed two times × 10 min in 0.1 M PBS 0.25% Triton-X. Sections were incubated for 5 min with 1:1000 DAPI in 0.1 M PBS 0.25% Triton-X. Sections were washed for 10 min in 0.1 M PBS and then mounted. In Fig. 7e, f, the 5xFAD × Thy1-GFPM mouse sections were stained for the mouse anti-human Aβ-antibody W0-2 (Millipore, Billerica, MA, USA) and as a secondary antibody for goat anti-mouse 488 (Invitrogen Alexa Fluor 488, A11029, Molecular Probes, Invitrogen, Eugene, OR, USA). First, the sections were pre-treated in 0.05 M citrate solution for 30 min (pH 6.0) at 80 °C. Endogenous peroxidase activity on sections was inhibited by incubation with 0.3% or 2% hydrogen peroxide in methanol. Non-specific antibody binding was blocked with 3% bovine serum albumin (BSA) or 10% NGS.

For immunocytochemistry, cells were washed three times with PBS, fixed with 4% PFA, 2% sucrose in PBS for 15 min, and then washed 3 times with PBS. Blocking, to reduce unspecific antibody binding, was done with 2% NGS, 1% BSA, and 0.1% saponin in PBS for 1 h. Cells were incubated with primary antibody (see Table 1) in PBS-T 3% NGS overnight at 4 °C. Cells were then washed with PBS-T for 5, 15, and 15 min and then incubated with secondary antibodies at 1:500 for 1 h in the dark. Cells were again washed with PBS-T 3 times for 5 min each and during the second wash 0.1% DAPI was added. Coverslips were then mounted on glass slides with slow fade gold anti-fade reagent (Life Technologies), dried in the dark overnight, and then sealed with Covergrip Coverslips Sealant (Biotum).

**Table 1:** Primary antibodies used in this study

Antibody	Epitope	Source	Identifier	Dilution
MOAB2	N-terminus Aβ	Abcam	ab126649	1:600 IHC
Beta-Amyloid Recombinant Rabbit Monoclonal (H31L21)	C-terminus Aβ42	Invitrogen	700254	1:600 IHC 1:300 ICC
NeuN	RbFox3	Millipore	ABN78	1:1000 IHC
GFAP	Glial fibrillary acidic protein	Sigma-Aldrich	G3893	1:500 IHC
Parvalbumin	Parvalbumin	Swant	Swant PV-25	1:500 IHC
MAP2	Microtubule Associated Protein2	Abcam	ab92434	1:1000 ICC
82E1	N-terminus of Aβ and C99	IBL	10323	1:700 WB
6E10	AA 1-16 of Aβ. Will detect Aβ, C99, APP and sAPPalpha	Biolegend	SIG-39320	1:1000 WB
Beta-actin	N-terminal end of Beta-actin	Sigma-Aldrich	A5316	1:4000 WB
OC	Amyloid fibrils and some oligomers	Merck	AB2286	1:5000 Dot blot
W0-2	AA residues 4-10 of Aβ	Millipore	MABN10	1:500 IHC

### Fluorescence microscopy

Confocal microscopy was performed with a Leica TCS SP8 laser scanning confocal microscope with LAS X software. A 5 × objective (HC PL Fluotar, NA 0.15, dry) was used for overview images, and image tile stitching was done by the LAS X software using the “Mosaic Merge” function. For all other images, a 40 × objective (HC PL APO CS2, NA 1.30, oil) was used. Imaris software (Bitplane) was used for image preparation. For quantification, imaging was done with a Nikon Eclipse 80i epifluorescence microscope. Images of the dentate gyrus of 5xFAD × Thy1-GFPM mice were taken with a Zeiss Axio Imager M2 microscope (Germany) with an AxioCam ERc5s camera using 10 × and 40 × objectives.

### Quantification of immunofluorescence

The Fiji 2 package of ImageJ was used for image analysis. To quantify intraneuronal Aβ, we used images of ErC stained with antibodies MOAB2 or H31L21 against Aβ and NeuN, to identify neuronal cell bodies. First, the NeuN channel was used as a mask (Fig. S1a, online resource) to quantify intraneuronal Aβ signal. Then, we applied a threshold, using the “moments” algorithm on the Aβ channel to remove background (Fig. S1a, online resource). The threshold was always identical between the injected and non-injected hemispheres. We then used the analyze particle command, excluding large particles, to quantify the percentage area of NeuN that also had Aβ-signal. To test the validity of this method we also performed quantification of intraneuronal Aβ in 6-week post-injection ErC by measuring total fluorescence and observed changes in the same directions as with analyzing particles but of a smaller magnitude (Fig. S1b, online resource). To quantify the number of NeuN-positive cells in stratum oriens, we used thresholding to define NeuN signal, manually defined only the stratum oriens as an area of interest, and then used analyze particles, excluding small particles, to count the number of NeuN-positive cells per unit of area. To quantify plaques, we defined an ROI based on DAPI-labeling and used thresholding to define plaques and then used analyze particles with small particles excluded (Fig. S2, online resource).

### Dot blot

Cell pellets were triturated in Tris-buffer containing 1% Triton-x and 1% protease inhibitor cocktail and then allowed to incubate for 30 min on ice. The solution was then centrifuged for 10 min at 10,000×*g* at 4 °C. The protein levels were quantified with BCA according to the manufacturer’s instructions (Pierce BCA protein assay kit, Thermo Fisher Scientific). The samples were then diluted to ensure identical protein levels in all samples. 1 µl of the samples were then applied onto nitrocellulose membranes, allowed to air-dry, and then washed three times for 15 min in PBS-T. Then, the membranes were blocked in 5% skim milk in PBS-T for 30 min, and then, primary antibody (OC at 1:5000) was applied overnight in 5% skim milk PBS-T at 4 °C. Then, membranes were washed 3 × 15 min in PBS-T, incubated for 1 h with secondary HRP antibody in 5% skim milk PBS-T at room temperature, and then again washed 3 × 15 min in PBS-T before development with ECL clarity solution (Bio-Rad).

### Western blot

Cells were lysed in 6% SDS and 1% β-mercaptoethanol in PBS, sonicated twice for 20 s at 20% amplitude (Branson SLPe model 4C15 sonifier), heated to 95 °C for 5 min, and centrifuged at 10,600×*g* for 10 min. Supernatants were mixed with SDS Sample Buffer (2 ×) (Thermo Fisher Scientific) with 0.8% β-mercaptoethanol, heated to 95 °C for 5 min, briefly centrifuged, and then loaded onto 10–20% or 16% Tricine Protein Gels with SeeBlue Plus2 Pre-stained Protein Standard (Thermo Fisher Scientific) used as a protein standard ladder. Actin was used as a loading control and all densitometric quantifications performed with Fiji 2 were normalized to actin.

### Statistical analysis

Statistical analysis was done using GraphPad Prism software 8 and 9 for Mac. All data sets were tested for normality using the Shapiro–Wilk test. All normally distributed data sets were assessed with *t* test or one-way ANOVA. One data set deviated significantly from normality, the “6 h low Aβ” in Fig. 5f; this may be spurious as we tested 17 data sets for normality (1–0.95^17^ = 0.58), nevertheless, we performed a non-parametric test for Fig. 5f (two-tailed Mann–Whitney test *p* = 0.019). All statistical tests used are noted in the figure legends, together with degrees of freedom (df), mean differences, standard error (SE or SEM) or standard deviation (SD), and number of replicates (*n*). All data are presented as individual data points with median and interquartile range and dashed lines indicate paired data.

## Results

### Prion-like cell lysate seeds plaques in vivo

**Fig. 1:**
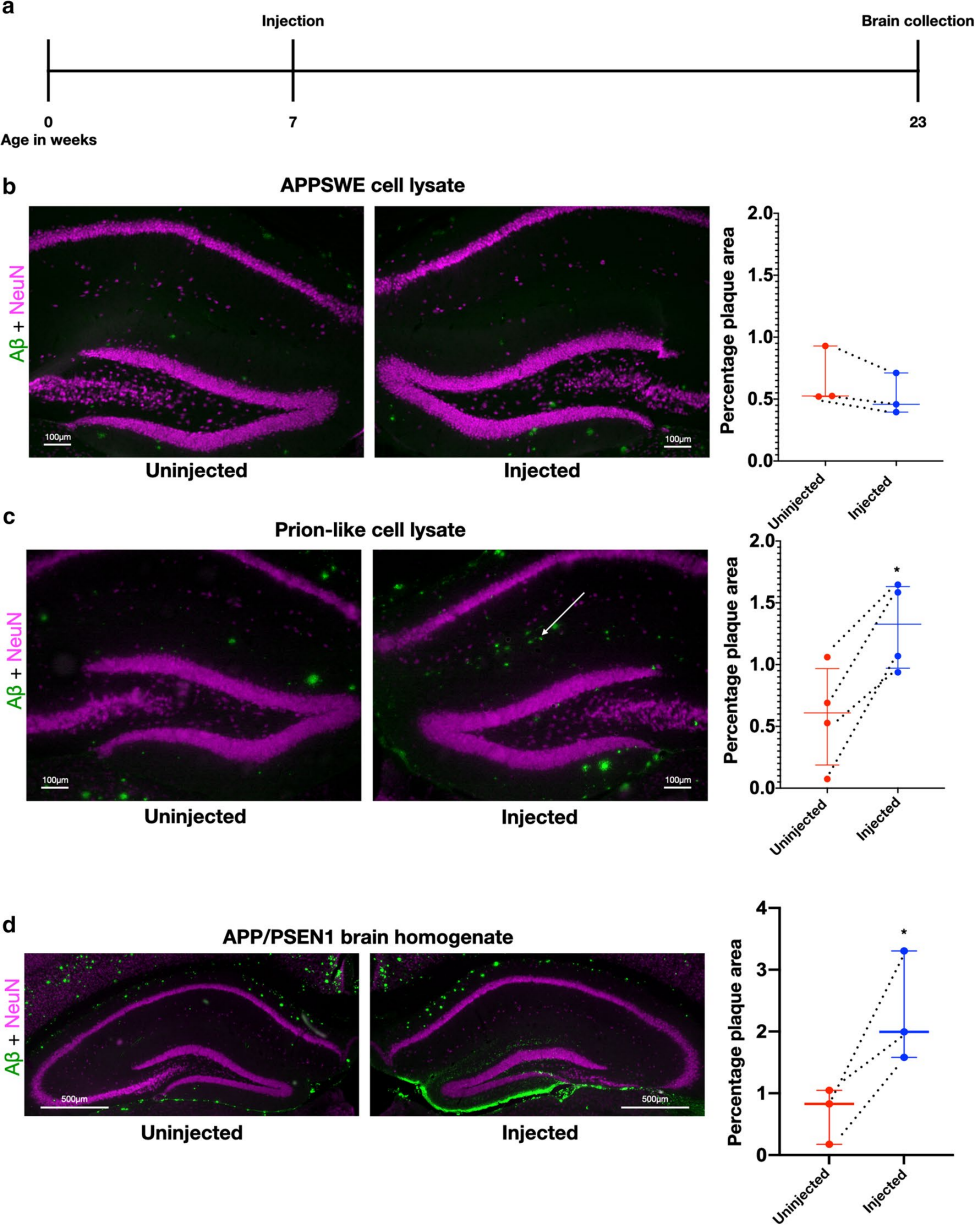
Prion-like cell lysate seeds plaques in vivo*.* **a** Timeline of unilateral injections into hippocampus of 7-week-old 5xFAD mice, followed by labeling at 23 weeks with MOAB2 (Aβ, green) and NeuN (neuronal cell bodies, magenta). **b** Unilateral injection in hippocampus of 5xFAD transgenic mouse with lysate of APPSwe N2a cells. Plaque quantification of the upper parts of the dentate molecular layer, and CA1 stratum lacunosum-moleculare and stratum radiatum (paired *t* test with Bonferroni correction, *p* = 0.18, df = 2, mean difference = 0.14 and SD of mean difference = 0.076, dashed lines indicate paired data and *n* = 3). **c** Mouse injected with cell lysate of prion-like cells. The induced plaques are found adjacent to the hippocampal fissure (arrow). Plaque quantification paired *t* test with Bonferroni correction, *p* = 0.026, df = 3, mean difference = 0.72 and SD of mean difference = 0.27, dashed lines indicate paired data and *n* = 4. **d** 5xFAD mouse injected with brain homogenate from a 21-month-old transgenic mouse with APP/PSEN1 mutations showing plaque seeding around the dentate gyrus on the injected side (paired *t* test, *p* = 0.0396, df = 2, mean difference = 1.61 and SD of mean difference = 0.57, dashed lines indicate paired data and *n* = 3)

The seeding of amyloid plaques by intracerebral injection of AD brain material into an AD-model mouse is well established [40]. However, the use of a homogenized brain, even if fractionated, makes it difficult to know what species of Aβ are responsible for the seeding and to determine if the Aβ came from an extra- or intracellular source. We previously reported on a prion-like clonal line of AD Swedish-mutant APP N2a neuroblastoma (SWE) cells that consistently produce and maintain intracellular aggregates of Aβ. We initially induced these aggregates by treating SWE cells with brain lysate from an AD-model transgenic mouse and then performed single-cell cloning to isolate lines with intracellular aggregates of Aβ [25]. We then showed that the lysates of our prion-like cells could induce Aβ aggregation in other SWE cells, demonstrating the presence of prion-like Aβ that could propagate in vitro in the cell line. In the present study, we show that these cell lysates are also capable of seeding in vivo*.* To this end, we performed unilateral intrahippocampal injections in 7-week-old 5xFAD transgenic mice with SWE cell lysate, prion-like SWE cell lysate, or APP/PSEN1 transgenic mouse brain lysate (positive control), using the contralateral hippocampus as a negative control. The mice were sacrificed 16 weeks post-injection and immunohistochemically processed for analysis (Fig. 1a; all antibodies used are in Table 1 in the Methods). Plaque quantification was done in the dorsal parts of the dentate molecular layer and CA1 stratum lacunosum-moleculare and stratum radiatum as shown in Fig. S2, online resource. No seeding was seen with the injection of control SWE cell lysate (Fig. 1b), while modest seeding was induced around the hippocampal fissure, in the molecular layer of the dentate gyrus, and in the stratum lacunosum-moleculare of CA1 by the injection of prion-like SWE cell lysate (Fig. 1c). In contrast, injection of APP/PSEN1 brain lysate caused robust seeding, including the area around the hippocampal fissure as in the brains injected with prion-like cell lysate. However, the strongest labeling was in the lower blade of the dentate gyrus, in particular in the outer molecular layer corresponding to the perforant path terminals of the lateral entorhinal cortex (LEC) (Fig. 1d). We thus demonstrate that an intracellular source of Aβ can seed amyloid deposition in vivo in a prion-like manner, albeit less robustly than brain extract*.*

### Seeded Aβ plaques follow anatomical pathways

**Fig. 2:**
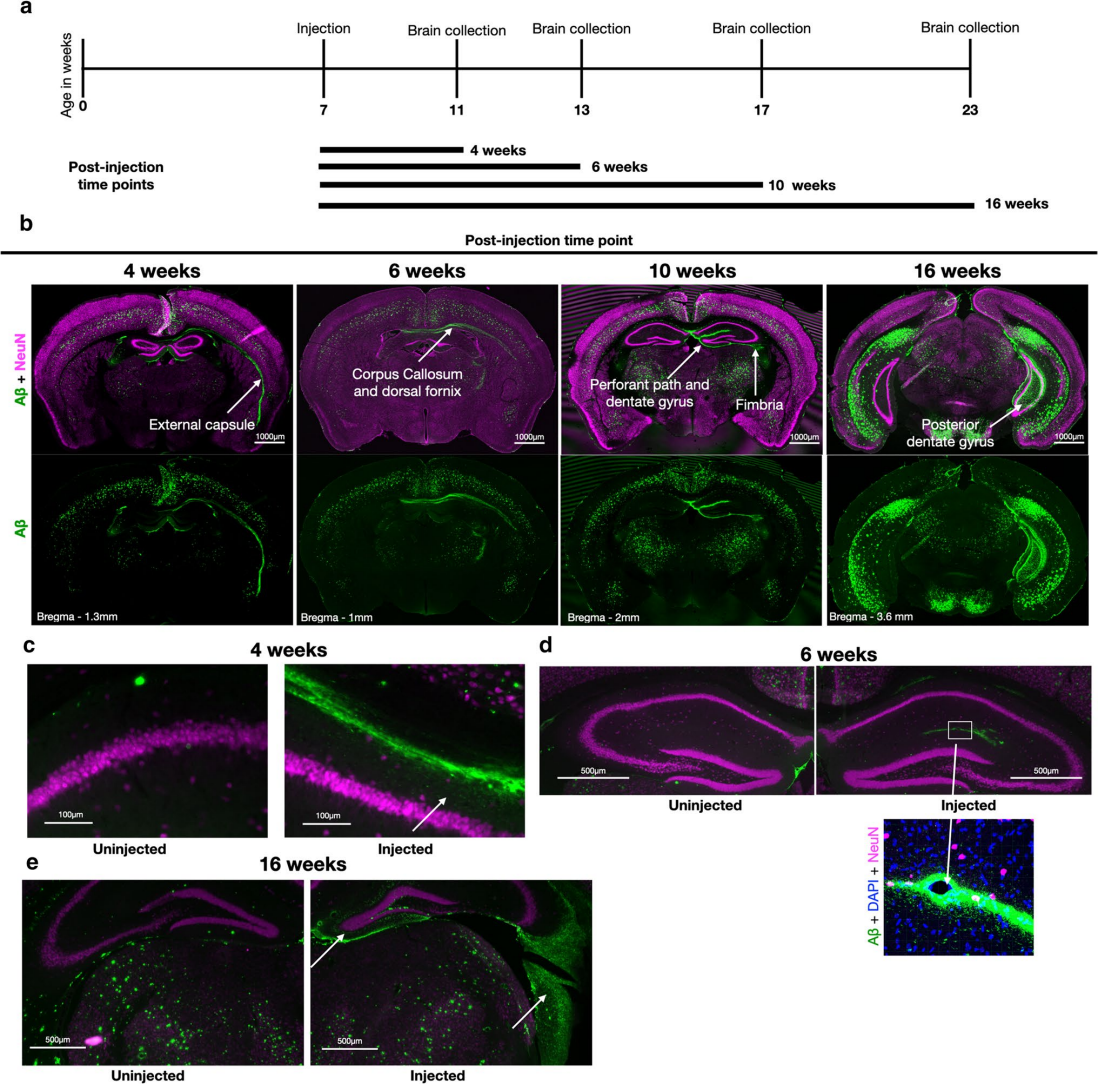
Seeded amyloid plaques follow anatomical projections. **a** Timeline of injections. Unilateral injection of the right hippocampus with 5 μl of 21-month-old APP/PSEN1 brain homogenate into 5xFAD mice at 7 weeks of age and sacrificed 4, 6, 10, or 16 weeks later. The brains in (**b**)–(**e**) are labeled with MOAB2 (green) for Aβ and NeuN (magenta) for neuronal cell bodies. Arrows show induced Aβ labeling. **b** Representative coronal brain sections of the different sacrifice time points. Note the preponderance of Aβ staining at early time points in/around white matter (external capsule, dorsal fornix, and corpus callosum) anterior to the injection. At later time points, the Aβ staining is also spread to posterior parts of the brain and then much reduced in external capsule and corpus callosum, while the fimbria of the hippocampus shows Aβ staining at 10 weeks and even more so at 16 weeks. **c** Small induced plaque-like structures (arrow) adjacent to white matter tracts can be observed already 4 weeks post-injection in the CA1 stratum oriens inferior to the corpus callosum. This is also evident adjacent to the external capsule. **d** 6 weeks post-injection, the most intense staining is around the hippocampal fissure, but it is also surrounded by small plaque-like structures (see insert) located in the CA1 and dentate gyrus molecular layers. **e** 16 weeks post-injection the typical location of induced plaques around the dentate gyrus (left arrow) is evident. There is also spread in the fimbria hippocampus (right arrow), a white-matter tract, and one of the main output paths of the hippocampus

Since the seeded Aβ seemed to associate with select anatomical pathways, the LEC perforant path, and the dorsal fornix of hippocampus, we more closely followed the progression of induced plaque pathology to obtain further insights into the cellular and anatomic mechanisms of seeded Aβ aggregation in vivo. APP/PSEN1 mouse brain homogenate was injected unilaterally into dorsal hippocampus of 7-week-old 5xFAD mice and sacrificed at 4, 6, 10, and 16 weeks post-injection (Fig. 2a). Already at 4 weeks post-injection, Aβ-induced seeding was evident in the injected side in the fibers of the alveus, corpus callosum, fornix, and the external capsule by the presence of immuno-fluorescently labeled Aβ (Fig. 2b). Notably, this initially seeded Aβ was consistently located somewhat anterior to the injection site (the injection was around − 2.5 mm Bregma). While at 4 weeks post-injection, the induced Aβ-signal was mostly confined to white-matter tracts, some induced Aβ could be seen in the brain parenchyma of CA1 stratum oriens adjacent to the alveus (Fig. 2c).

To ascertain what was the original injection material and what were induced plaques, we also injected WT mice with brain homogenate mixed with India ink. We sacrificed these mice 1, 4, and 6 weeks post-injection and observed India ink and human Aβ staining in the anterior parts of the external capsule and some speck-like labeling in the corpus callosum (Fig. S3, online resource). Notably, the Aβ staining always co-localized with the India ink and was faint in WT compared to 5xFAD mice, got fainter with time in the WT mice, and was not evident outside the aforementioned white-matter tracts. Thus, a small part of the injected Aβ in the white-matter tracts is likely from the original injection material in the 5xFAD mice. In contrast, the Aβ in the underlying grey matter outside the white-matter tracts is induced.

At 6 weeks post-injection, induced Aβ in 5xFAD mice was apparent in the injected side of the dorsal fornix and became more pronounced in the corpus callosum, alveus, and external capsule (Fig. 2b). At this time-point, we also observed robust plaque-like structures outside of the white-matter tracts, primarily in the CA1 stratum oriens underlying the corpus callosum. Interestingly, in one animal in this 6-week post-injection group, we also observed Aβ plaques in the border zone of the outer molecular layer of the dentate gyrus and the stratum lacunosum-moleculare of CA1, i.e., surrounding the hippocampal fissure (Fig. 2d). At 10 weeks post-injection, induced Aβ aggregates were found mainly in the dentate gyrus (Fig. 2b), although one mouse exhibited aggregates in the corpus callosum in addition to spread in the hippocampus (Fig. S4, online resource). At 16 weeks post-injection, all mice displayed prominent induced plaques concentrated in the molecular layer of the dentate gyrus that expanded into more posterior areas, along with aggregates/spread in the fimbria of the hippocampus (Fig. 2b, e) and the stratum oriens of CA3. Interestingly, none of the 16 weeks post-injection mice displayed prominent Aβ-aggregates in the fornix, alveus, or the external capsule, supporting the conclusion that such aggregates that were observed in these fiber tracts after shorter incubation times represent a transient phenomenon, peaking at 6–10 weeks post-injection (Fig. S5, online resource).

### Loss of NeuN in stratum oriens

**Fig. 3:**
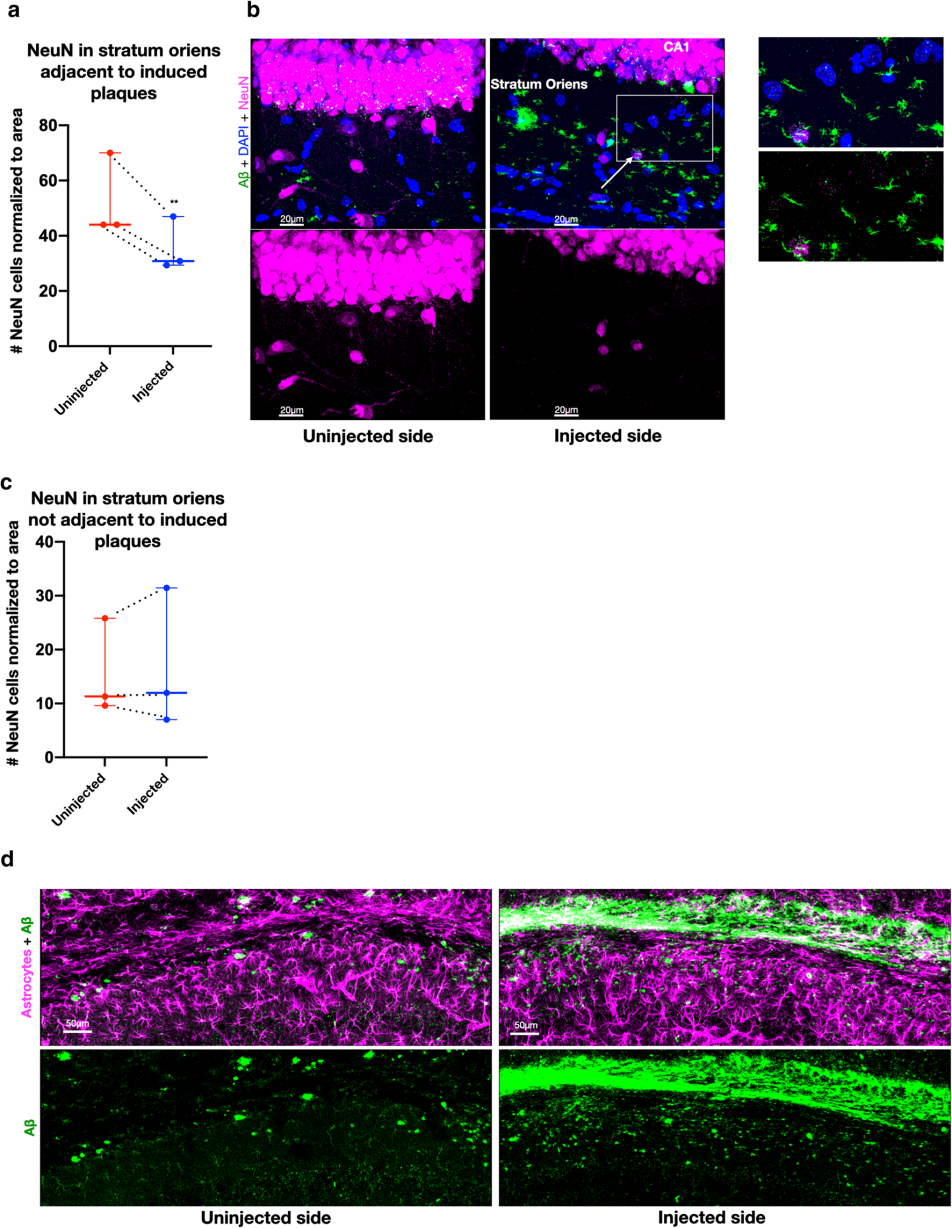
Injected side with loss of NeuN and increased amyloid plaques. **a** Number of NeuN cells in stratum oriens in mice 6 weeks post-injection where plaques were induced. There was a 32% decline of NeuN-positive cells adjacent to induced plaques in the stratum oriens compared to the uninjected side (two-tailed ratio-paired *t* test, *p* = 0.0015 df = 2, geometric mean of ratios = 0.68 and SD of log-ratios = 0.011, dashed lines indicate paired data and *n* = 3). **b** Confocal 40 × image of CA1 stratum oriens in 5xFAD mouse 6 weeks post-injection. Note the weaker NeuN staining in the injected side (arrow) and cells with remnants of NeuN (box magnified to the right). MOAB2 (green) for Aβ, NeuN (magenta) for neuronal cell bodies, and DAPI (blue) for all cell bodies. **c** No decline of NeuN-positive cells not adjacent to induced plaques in stratum oriens of the injected side (two-tailed ratio-paired *t* test p = 0.91, df = 2, geometric mean of ratios = 0.98 and SD of log-ratios = 0.12, dashed lines indicate paired data and *n* = 3). **d** Small plaque-like structures in the CA1 stratum oriens in the injected side 6 weeks post-injection. These Aβ structures (MOAB2, green) do not co-localize with astrocytes (GFAP, magenta). Maximum intensity confocal image 40 ×

Due to the early appearance of seeded Aβ aggregates in parts of the stratum oriens of CA1, we conducted a closer examination of the neurons in that area. Remarkably, there was a decrease in NeuN labeling in this layer near to where the injection had seeded Aβ aggregates compared to the uninjected side (Fig. 3a, b). There was also greater heterogeneity in the intensity of NeuN labeling near induced Aβ aggregates with numerous nuclei that were weakly NeuN-positive (Fig. 3b). The loss of NeuN with intact DAPI suggests damage but not death of the neurons [11]. In the parts of stratum oriens of the injected side where plaques had not been induced, there was no decline of NeuN-positive cell labeling (Fig. 3c). The neurons in CA1 stratum oriens are interneurons and early loss of interneurons has been described in AD and the 5XFAD mouse [8]. We noted that the induced plaque-like labeling in the stratum oriens represented smaller deposits of aggregated Aβ than typical larger amyloid plaques (Fig. 3d). This wisp-like Aβ labeling, which among other markers was negative for GFAP, may be consistent with dystrophic neurites, which have been shown to accumulate the earliest small Aβ aggregates by immuno-EM [33, 35].

### Dynamic intraneuronal Aβ changes in entorhinal cortex layer II and decreased intraneuronal Aβ in CA1 pyramidal neurons near plaques

**Fig. 4:**
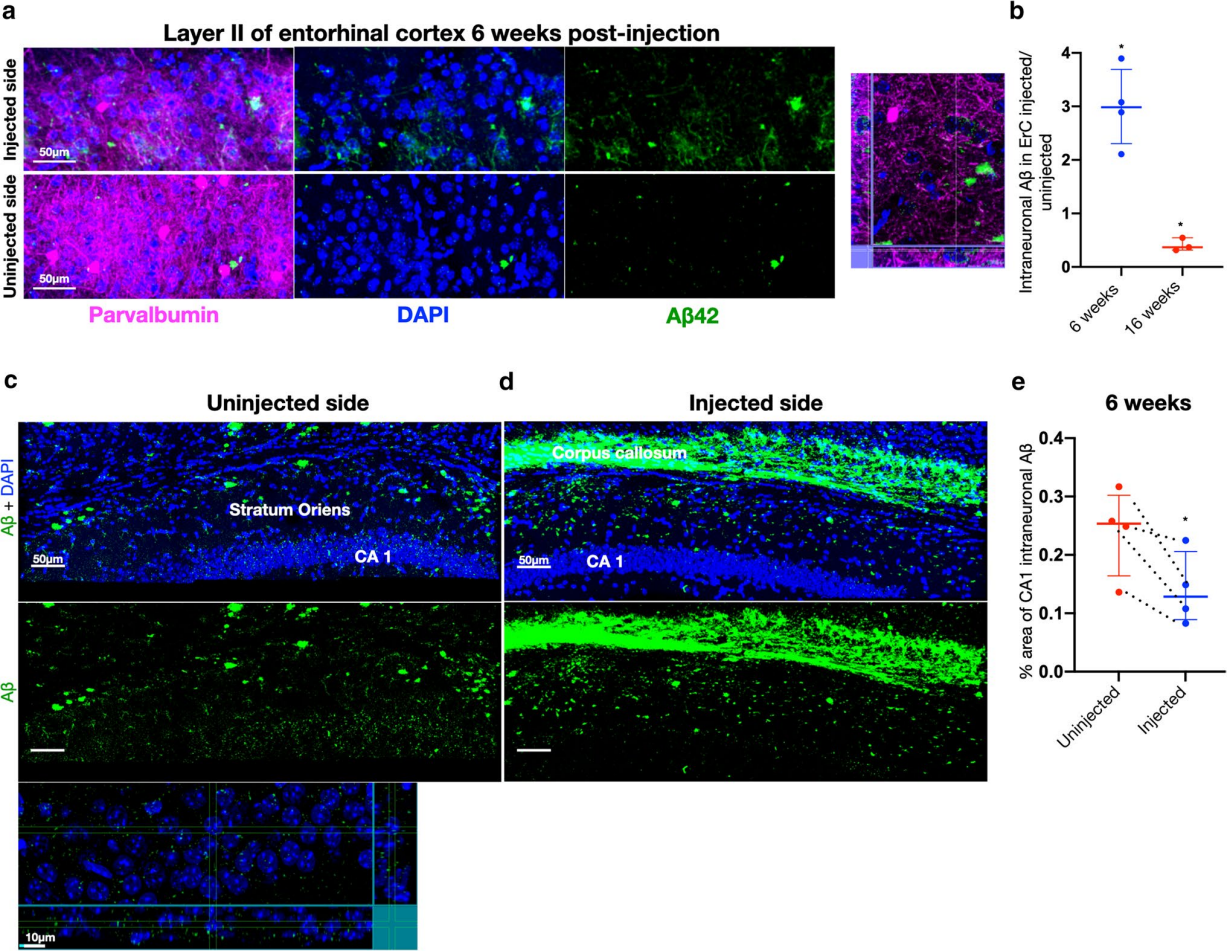
Dynamic intracellular Aβ changes in entorhinal cortex and decreased intracellular Aβ in CA1 pyramidal neurons near plaques. **a** Representative 40 × confocal image of the layer II entorhinal cortex (synaptically connected to the hippocampus) 6 weeks post-injection, as well as XYZ slice to show intracellular Aβ. Parvalbumin (magenta), Aβ42 antibody H31L21 (green), and DAPI (blue). **b** Quantification of the ratio of intraneuronal Aβ in ErC layer II in the injected to uninjected side. 6 weeks post-injection, there was significantly more intraneuronal Aβ (Aβ co-localizing with NeuN) in the injected side, while after 16 weeks, the pattern was reversed (one-sample *t* test against mean of 1 with Bonferroni correction for multiple comparisons, *p* = 0.0244, df = 3, mean = 2.99, discrepancy = 1.99, SD of discrepancy = 0.73 and *n* = 4 for 6 weeks and *p* = 0.0268, df = 2 and mean = 0.41, discrepancy = 0.59, SD of discrepancy = 0.12 and *n* = 3 for 16 weeks). **c** More Aβ is evident inside the pyramidal neurons of CA1 in the uninjected compared to the injected (**d)** side 6 weeks post-injection. Confocal 40 × maximum intensity image and XYZ section to show intracellular Aβ. **d** In contrast, in the injected side, we see strong Aβ labeling in the corpus callosum, more small plaque-like structures in the CA1 stratum oriens and lower levels of Aβ in the pyramidal neurons compared to the uninjected side (**c**); MOAB2 (green) for Aβ and DAPI (blue) for nuclei. **e** Ratio of the amount of CA1 intraneuronal Aβ in the uninjected to injected side. There is an approximately 40% reduction of Aβ in the CA1 pyramidal neurons on the injected compared to uninjected side (two-tailed ratio-paired *t* test *p* = 0.047, df = 3, geometric mean of ratios = 0.57 and SD of log-ratios = 0.15), dashed lines indicate paired data and *n* = 4). Note that **c** and **d** are the same images as in Fig. 3c but now showing DAPI and not GFAP to focus on intracellular Aβ in CA1

In the dentate gyrus, much of the Aβ seeding corresponded to the terminal fields of afferents originating in LEC layer II neurons (a major component of the perforant path). We therefore further examined the cell bodies of LEC layer II neurons in brains that had Aβ seeding associated with the perforant path axonal terminals (Fig. 4a). Interestingly, the levels of intraneuronal Aβ in soma of LEC layer II were increased in the injected versus uninjected side at 6 weeks post-injection but then decreased in the injected versus uninjected side at 16 weeks post-injection (Fig. 4b). There was no difference in total Aβ immunofluorescence of the LEC between 6 and 16 weeks in the uninjected sides, which however is a statistically less powered comparison, since one then compares between different mice and sections rather than internally with the contralateral hemisphere of the same section. This is consistent with the Aβ of the injected LEC changing between 6 and 16 weeks, but not the uninjected side.

We also noted that the levels of Aβ in the cell bodies of the CA1 pyramidal neurons were decreased in the injected side, where plaques had been induced in the adjacent stratum oriens (Fig. 4c, d, e). One possibility was that the pyramidal cells were losing their intracellular Aβ to the nearby induced plaques. The induced plaques are located near the cell bodies and basal dendrites of the CA1 neurons. Another possibility is that Aβ is redistributed from CA1 cell bodies to processes in the injected side. It has been shown that primary AD-transgenic neurons accumulate aggregated Aβ in their processes with time in culture [33].

### Extra- and intracellular pools of Aβ exist in an equilibrium that regulates Aβ production

Thus far, our in vivo work showed that intracerebral injection of prion-like seeds of Aβ not only induced plaques, but also affected intracellular (IC) Aβ. Furthermore, the decline of IC Aβ in CA1 pyramidal neurons suggested a possible equilibrium between IC and interstitial fluid (ISF) Aβ. In our previous work with the prion-like Aβ N2a cell line, we noted changes in APP processing with the accumulation of IC Aβ, namely increased β-cleavage but no change in α-cleavage [25]. For these reasons, we next sought to investigate how IC aggregation of prion-like Aβ might affect APP processing and the equilibrium between extracellular (EC) and IC Aβ. To this end, we manipulated levels of IC and EC Aβ in the prion-like cells and parent SWE N2a cells.

**Fig. 5:**
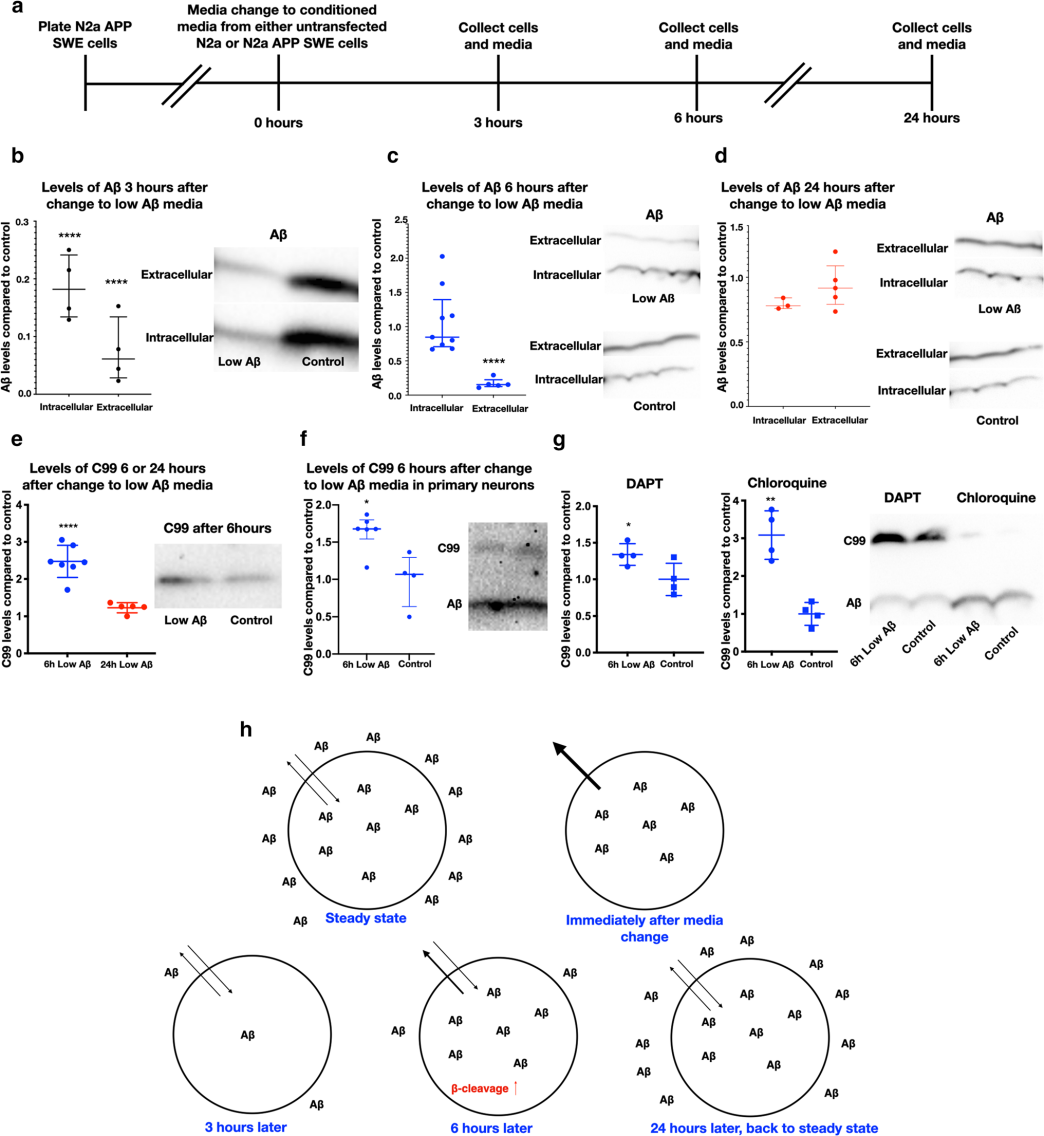
Extra- and intracellular pools of Aβ exist in an equilibrium that regulates Aβ production (full blots and graphs can be seen in Fig. S6, online resource). **a** Timeline of experiments. **b** 3 h after change to low Aβ media, there is 80% less intracellular Aβ and 90% less extracellular Aβ (one-way ANOVA Sidak’s test for multiple comparisons, *p* < 0.0001 for both, df = 12 for both and mean difference = 0.81 for intracellular Aβ and 0.93 for extracellular, SEM = 0.077 for both and *n* = 4 for all). **c** 6 h after media change to low Aβ media the intracellular pool of Aβ has recovered to control levels, while the extracellular levels remain low at 80% below control (two-tailed unpaired *t* test, *p* < 0.0001, df = 8, mean difference = 0.83,SEM = 0.059 and *n* = 5). **d** 24 h after media change, there are no longer any significant differences in Aβ; the equilibrium has returned. **e** 6 h after change to low Aβ media, APP C99 is about 2.5 times higher than control, while at 24 h, there is no difference (one-way ANOVA Sidak’s test for multiple comparisons, *p* < 0.0001 and *p* = 0.53, df = 20 for both, mean difference = 1.47 and 0.21 and *n* = 7 and 5). **f** Primary neurons also increase C99 6 h after change to low Aβ media (two-tailed non-parametric test, Mann–Whitney *p* = 0.019, median difference = 0.61 and *n* = 6 and 4). **g** APP C99 was increased about 30% with low Aβ media even when treated with gamma-secretase inhibitor DAPT (two-tailed unpaired *t* test, *p* = 0.0429, df = 6 and mean difference = 0.34) and by about 200% when treated with lysosomal inhibitor chloroquine (two-tailed unpaired *t* test, *p* = 0.0011, df = 6, mean difference = 2.08 and *n* = 4 for both). **h** Model of how extra- and intracellular Aβ change in N2a APP Swedish cells after change to low Aβ media

We first examined SWE cells without any prion-like induced IC Aβ. To remove the pool of EC Aβ, we replaced the media with conditioned media (CM) from untransfected N2a cells. We then performed Western blots and densitometric quantification, adjusted to actin, to assess levels of Aβ, the APP C-terminal fragment (CTF) C99, and full-length APP (flAPP) in cells and media at 3, 6, or 24 h after the media change (Fig. 5a). After 3 h, there was a greater than 80% decrease of IC Aβ (Fig. 5b), which could be accounted for by secretion of the IC Aβ. At the 6-h time-point, the IC Aβ levels had recovered to the same levels as control, while the EC levels were still low (Fig. 5c). Finally, 24 h after changing the media, the IC and EC Aβ levels reached levels similar to the controls (Fig. 5d). These changes are consistent with a prior report, showing that depletion of the EC Aβ pool is followed by depletion of the IC pool, after which the latter recovers first [24]. Interestingly, we also observed significantly increased C99 levels 6 h after the media change with untransfected N2a cell CM (Fig. 5e). C99 is a fragment of flAPP that is generated by β-secretase, and further cleavage of C99 by γ-secretase produces Aβ. In contrast to the C99 levels after 6 h, C99 levels were not altered at 3 h (data not shown) nor 24 h after media change (Fig. 5e). Finally, under no condition did we observe any significant change in the levels of flAPP. We also performed the 6-h low Aβ CM experiment in primary neurons and saw that C99 was similarly increased (Fig. 5f).

To examine whether decreased lysosomal degradation of C99 rather than increased β-cleavage of flAPP could cause the C99 increase with low CM Aβ, we repeated the media change experiment above but pre-treated the cells with the lysosomal inhibitor chloroquine. If decreased lysosomal degradation was the reason for increased C99 after 6 h of low Aβ media, then blocking degradation should eliminate the C99 difference between cells treated with low Aβ compared to baseline media (control). As expected, chloroquine increased Aβ in all conditions. However, there were more C99 in the low EC Aβ condition compared to control (Fig. 5g), supporting the conclusion that decreased lysosomal degradation was not the primary cause of the elevated C99 with low Aβ media. Further supporting this point, cells treated with the γ-secretase inhibitor DAPT, which inhibits the production of Aβ from C99, thereby increasing C99, still had elevated levels of C99 after 6 h of low Aβ media compared to control media (Fig. 5g), indicating that lower γ-cleavage is also not the primary reason for the increased levels of C99. Thus, it is likely that β-cleavage of APP is upregulated in response to low levels of EC Aβ. We summarize our findings from our Aβ equilibrium studies in N2a SWE cells in a schematic (Fig. 5h).

The addition of synthetic Aβ has been reported to increase Aβ production via increased β-cleavage in a cell line overexpressing APP [45]. The addition of exogenous Aβ will increase both EC and IC Aβ. To expand on this finding, we treated SWE cells with 1 µM of synthetic Aβ1-42 for 3, 6, or 24 h. Three hours after adding 1 µM of EC Aβ, there was no change in the levels of C99. However, 6 h post-treatment, there was a significant increase in C99. These levels were reduced by the 24-h time-point, but were still much higher than under control conditions (Fig. S7b, online resource). Notably, when measured 24 h after addition of Aβ1-42, the IC Aβ remained around 30 times higher in the treated cells compared to control (Fig. S7c, online resource). Taken together, these findings underscore the equilibrium of the extra- and intracellular pools of Aβ and show that both reduced and elevated EC Aβ can stimulate β-cleavage to elevate Aβ production.

### Intracellular aggregation of prion-like Aβ disrupts the equilibrium between extra- and intracellular Aβ

**Fig. 6:**
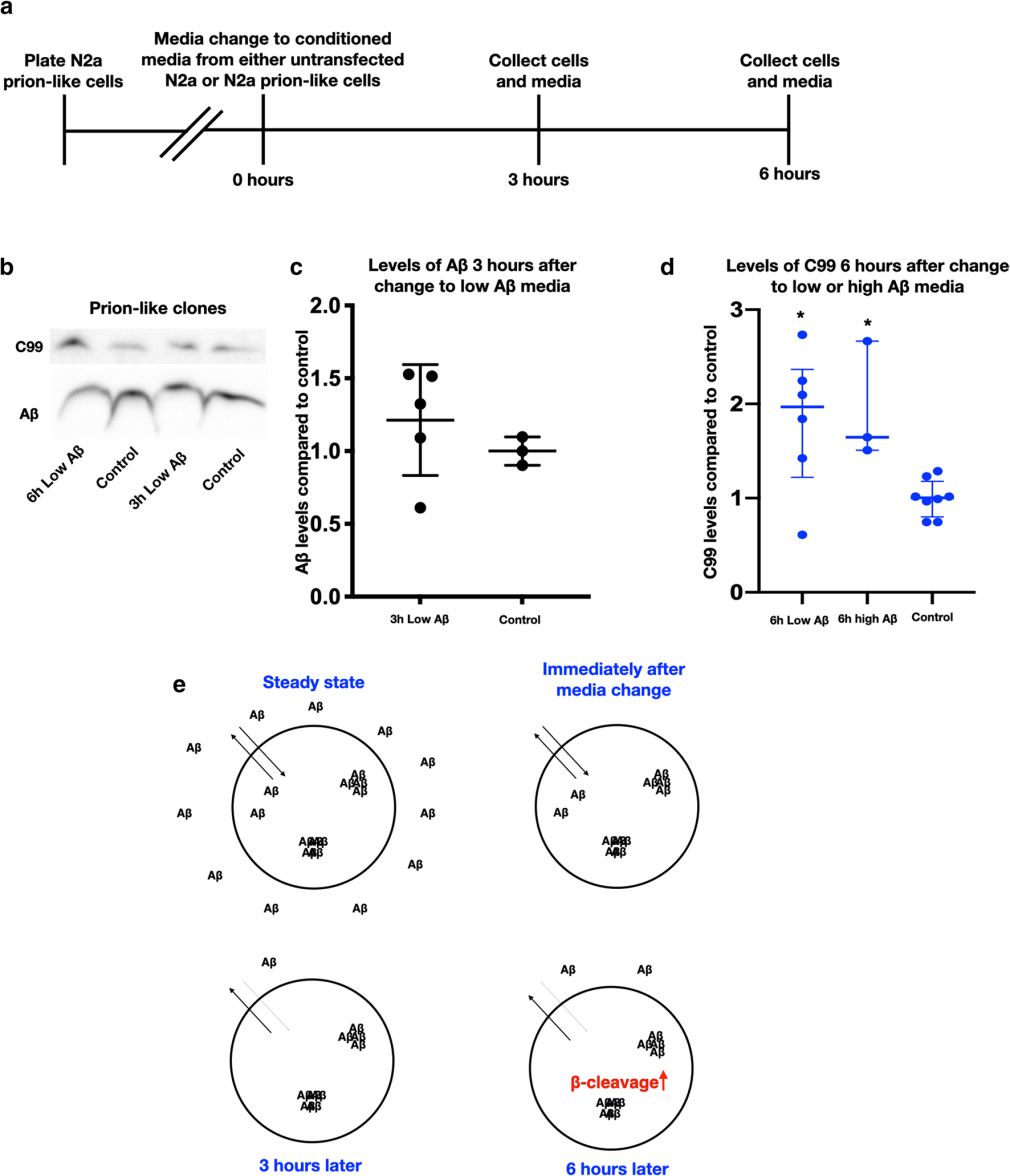
Intracellular aggregation of Aβ in prion-like clones disrupts the equilibrium between extra- and intracellular Aβ. **a** Timeline of experiments. **b** Representative immunoblots of prion-like clones and how levels of Aβ and C99 change when the media are changed to low Aβ media (media from untransfected N2a cells). **c** Densitometric quantification of intracellular Aβ in prion-like clones showing no change after 3 h of low Aβ media. This is in contrast to the 80% decline in intracellular Aβ in normal N2a APPSWE cells seen in Fig. 5b. **d** Densitometric quantification of C99 showing that even in prion-like clones C99 is increased in response to low and high Aβ (one-way ANOVA Sidak’s multiple comparisons test, for low Aβ *p* = 0.0214, mean difference = 0.82 with an SE of 0.35, *n* = 6, df = 14, *n* = 6 and for high Aβ *p* = 0.0361, mean difference = 0.94 with an SE of 0.28 df = 14 and *n* = 3). **e** Model of how extra- and intracellular Aβ changes in prion-like cells after the change to low Aβ media

In N2a SWE cells, reducing EC Aβ quickly lowered IC Aβ levels (Fig. 5b). Remarkably, when we repeated these experiments with our prion-like cell line, which have intracellular aggregates of Aβ (Fig. 6b), the changes in IC Aβ levels due to low Aβ media seen in the parent SWE line were no longer present. At the 3 h post-media change time-point, CM from untransfected cells (low Aβ) did not reduce IC Aβ in the prion-like cells (Fig. 6b, c). Notably though, C99 was still elevated after 6 h of low EC Aβ (Fig. 6b, d), indicating that low EC Aβ drives the increase in C99 and not low IC Aβ. It should also be noted that the prion-like cells constitutively have 3–4 times the amount of IC Aβ and up to 10 times higher levels of C99 compared to the parent SWE cells [25]. Thus, in our cell model, under conditions of both high IC Aβ and low EC Aβ, the production of Aβ could be permanently upregulated. We summarize our findings for this altered Aβ equilibrium in prion-like cells in a schematic drawing (Fig. 6e).

### Induced Aβ aggregation in primary neurons and Aβ redistribution from soma to terminals

**Fig. 7:**
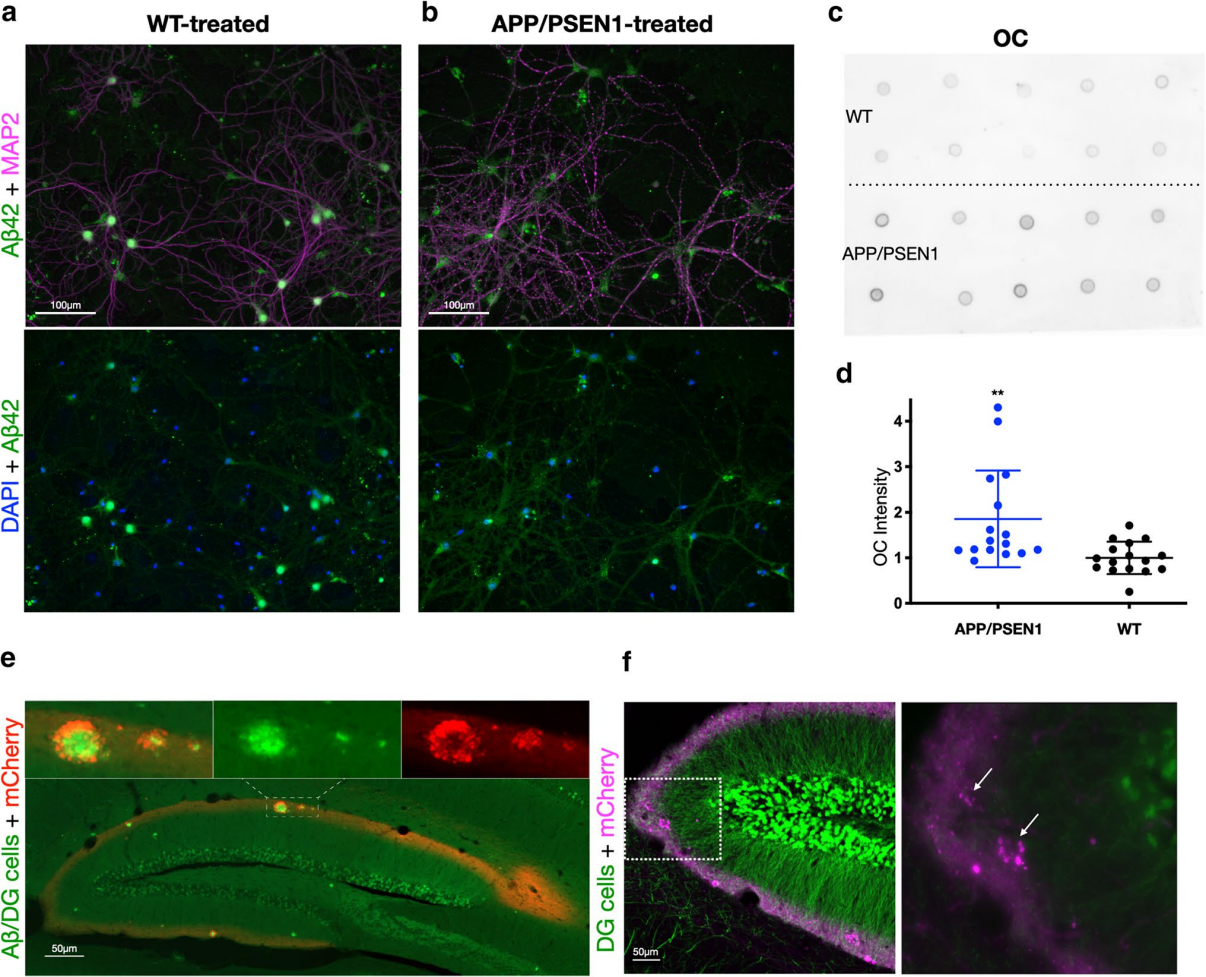
Induced Aβ aggregation in primary neurons and Aβ redistribution from soma to terminals. **a** APP/PSEN1 primary neurons that have been treated with 100 k×*g* supernatant of WT brain. Here, Aβ labeling is more evident in the neuron soma. **b** APP/PSEN1 primary neurons treated with 100 k×*g* supernatant of APP/PSEN1 brain; note the beading of the dendrites (MAP2) and the more evident Aβ labeling in neurites compared to neuron soma. **c** Dot blot of top WT treated neurons and bottom APP/PSEN1-treated neurons, 0.46 μg of total protein per dot. OC is a confirmation-specific antibody against fibrils and fibrillar oligomers. **d** Densitometric quantification of antibody OC intensity showing 86% more OC signal from primary neurons treated with APP/PSEN1 brain ultra-centrifugate compared to WT (two-tailed unpaired *t* test, *p* = 0.0047, df = 30, difference between means = 0.86, SEM = 0.28 and *n* = 16). **e** A dentate gyrus (DG) section of a 5xFAD × Thy1-GFPM mouse injected with AAV-mCherry into the lateral entorhinal cortex and stained with Aβ antibody W02. The endogenous GFP in DG granule cells is seen as mossy green, while Aβ (antibody W02) appears as bright green. The red fluorescence is limited to the outer molecular layer, the main projection zone of the lateral entorhinal cortex. Insets (above): three magnified amyloid plaques (Aβ, green) of different sizes with dystrophy-like labeling, surrounded by red dystrophic axon terminals (mCherry) of entorhinal layer II neurons; merged at left, Aβ (green) alone in center and mCherry (red) alone at right. For those with decreased ability to distinguish between red and green, see Fig. S8B, online resource. **f** An adjacent section from the same mouse without Aβ staining and quenching of the endogenous GFP fluorescence. Magnified box to the right: Dystrophic axons (magenta) surround two amyloid plaques (arrows). Note the absence of green staining in the plaque centers

We next examined whether we could seed Aβ aggregation in primary neurons and how it affects the intraneuronal distribution of Aβ. To this end, we treated AD-transgenic primary neurons in culture with APP/PSEN1 transgenic mouse brain lysate. As controls, we treated transgenic primary neurons with brain lysate from either WT or APP KO mice and WT neurons with brain lysate from APP/PSEN1 or APP KO mice. While our previous study had shown that N2a cells could tolerate 3000×*g* supernatant from mouse brain lysate [25], primary neuronal cultures were more sensitive and died after the addition of this lysate regardless of whether it was from WT, APP KO, or APP/PSEN1 brain. To overcome this issue, we used brain lysate supernatant obtained from ultracentrifugation at 100,000×*g*. While this process removes the vast majority of Aβ, it has been shown that this fraction can still induce significant seeding in vivo [7]. We, therefore, treated APP/PSEN1 mouse primary neurons with 0.5% of media volume of 100,000×*g* supernatant from 21-month-old WT, APP KO, or APP/PSEN1 mouse brain at 12 and 19 days in vitro (DIV) with the final added Aβ concentration in the picomolar range. The primary neuron cultures were then either fixed or collected and pelleted at 28 DIV. To visualize potential seeding, we performed immunofluorescence with antibodies against Aβ42 and MAP2 (Fig. 7a, b). In the cultures treated with APP/PSEN1 brain lysate, we observed increased Aβ42-labeling in processes but reduced labeling in the soma. We also noted beading of the MAP2 labeling, indicating dendritic beading, which has been associated with excitotoxicity, dystrophic neurites, and AD [10, 30, 38]. In contrast, WT neurons treated with APP/PSEN1 brain lysate did not show dendritic beading or redistribution of Aβ42 (Fig. S7, online resource). We next performed dot blots on the collected pellets of primary neurons with the antibody OC (Fig. 7c) that reacts against aggregated amyloid proteins [16]. Remarkably, there was almost a twofold increase in OC labeling in cell lysates from the AD-transgenic cultures treated with APP/PSEN1 mouse brain lysate compared to controls (Fig. 7d). Taken together, we provide evidence that a minute amount of brain-derived Aβ can induce aggregation of Aβ in primary neurons, appears to redistribute Aβ from soma into processes, and causes dendritic beading.

Finally, to determine whether a similar redistribution to terminals occurs in vivo, we injected adeno-associated virus (AAV) with a vector encoding mCherry into the LEC of 5xFAD mice crossed with transgenic mice that express GFP in dentate granule cells. We observed distended mCherry-positive LEC layer 2 axonal terminals within developing plaques in the outer molecular layer of the dentate (Fig. 7e); this is in line with evidence showing the importance of axonal transport from LEC to plaque formation in the outer molecular layer of the dentate by perforant path lesioning [20, 31]. Furthermore, different sizes of plaques were evident, with the size proportional to the number of distended mCherry-positive LEC axonal terminals present, consistent with a progression in plaque size depending on the associated number of dystrophic neurites (Fig. 7e). The insets highlight these potential stages of plaque formation: in the largest plaque, Aβ labeling is seen in the center (core) but also co-localizing within mCherry-positive LEC axon dystrophies just internal to mCherry-positive but Aβ negative outer dystrophies, while the medium-sized plaque shows no core and mostly mCherry-positive/Aβ-negative dystrophies except in the middle (Fig. 7e, inset). Even a single mCherry dystrophy is evident, which, however, only has minimal Aβ labeling. In contrast, dentate granule cell dendritic GFP is not pronounced in these axonal dystrophy-derived plaques, consistent with the absence of intracellular Aβ accumulation in the dentate granule cells (Fig. 7f).

## Discussion

The pathogenesis of AD begins long before clinical dementia. A decline of Aβ42 levels in CSF can be seen 20 years before the onset of dementia. This decline occurs even before the detection of amyloid plaques by brain amyloid imaging [2, 26], supporting the concept that pre-plaque cellular changes involving Aβ take place early in the disease. We propose that the formation of a prion-like intracellular seed of Aβ may be one of the earliest changes in AD. In this study, we show that an intracellular source of aggregated Aβ induces plaque formation in a susceptible mouse. Furthermore, we perform intracerebral injection of AD-model mouse brain homogenate into the 5xFAD transgenic mouse, replicating the previous studies showing induction of plaques in hippocampus [48] and further noting significant Aβ spread in white-matter tracts, although higher resolution ultrastructural imaging is needed to definitively determine whether this is within and/or around white-matter tracts. More importantly, we examine how intracellular Aβ is affected in anatomically connected areas. We find that before induced plaques form in fields associated with the lateral perforant path terminals, the connected LEC layer II neuron soma first gain intracellular Aβ but then lose it following robust induction of plaques at their terminals. The LEC is located far from the injection site, but is massively connected with the hippocampus via the perforant path, which forms the cortical input to the outer molecular layer of the dentate gyrus [44]. Thus, the effects on intracellular Aβ are consistent with early neuronal transport of prion-like Aβ. The LEC is among the earliest affected brain areas in AD. It is an early site of tau pathology (hyperphosphorylated tau and neurofibrillary tangles) but also an area with early intraneuronal Aβ [9, 41], particularly in reelin-positive layer II projection neurons [17]. In both mice [24, 43] and humans [9, 22], it has been shown that intracellular Aβ in AD-vulnerable neurons precedes plaque formation but subsequently decreases with extensive plaque formation. We also detected a decline in Aβ in pyramidal neuron soma of CA1 following plaque induction in the adjacent stratum oriens, where CA1 neurons project both dendrites and axons. Thus, the introduction of prion-like Aβ not only accelerates plaque formation but also accelerates the intracellular Aβ changes seen in AD. We show that minute amounts of brain-derived Aβ induce Aβ aggregation and what appears to be a redistribution of intracellular Aβ from soma to processes, as well as neuritic beading in AD mutant primary neurons. Notably, neuritic beading has been reported after treating WT primary mouse neurons with 1 µM oligomeric Aβ42 [30]. Here, we induced neuritic beading by treating AD-transgenic primary neurons with picomolar amounts of Aβ from 100,000×*g* ultracentrifuged 5xFAD brain extract. It is likely that brain-derived Aβ is more toxic to neurites than synthetic Aβ oligomers, but this may also be compounded by the enhanced toxicity of Aβ in a susceptible host that overproduces (human) Aβ, likely due to prion-like mechanisms. Thus, even in primary neurons, the introduction of prion-like Aβ may accelerate the intracellular Aβ changes seen in AD.

It should be noted though that our in vivo work is with the 5xFAD mouse model of AD, which is an aggressive AD model with five AD causing mutations, something that is not seen in a human. However, induction of plaques via intracerebral injection of AD brain material has been shown in several, including less aggressive, AD models both in mice and rats [21, 29, 48]. Another limitation in our study pertains to the quantification of intraneuronal Aβ. Fluorescent microscopy is not optimal for precise quantification. We quantified signal from Aβ antibody MOAB2 co-localizing with neuronal soma marker NeuN as a proxy for intraneuronal Aβ and confirmed that it was intracellular with confocal microscopy. Fluorescent labeling inevitably has background and we, therefore, performed thresholding followed by particle analysis with Fiji 2. As an alternate method, we performed total fluorescence analysis, which yielded differences of a smaller magnitude; this is expected given the inclusion of background signal, although the results were similar. Using automated algorithms, we reduced bias and by virtue of our unilateral injection we could pair data, which reduces variability and increases statistical power. Another limitation is that while we noted the loss of NeuN, we did not assess neuron cell death. Reduced NeuN associated with preserved DAPI signal can be seen with neuron damage even without loss of neurons [11]. However, loss of interneurons in CA1 hippocampus layers is seen early in 5xFAD mice [8] and Aβ aggregation in CA1 interneurons was previously associated with early plaque formation in 5xFAD mice [3].

Our data support an alternate origin of plaques in AD from what is traditionally described in the literature, wherein Aβ pathology has been posited to develop in the extracellular space. Early plaque formation induced by injection of exogenous Aβ-containing extracts led to the first new plaques in the stratum oriens of CA1 hippocampus, where we also saw a decline in NeuN-positive neurons. Such an association between increased Aβ and declining NeuN is consistent with plaques originating from neuronal cell bodies, as has been described previously [3, 6, 9, 27]. The location of these neurons in stratum oriens implicates inhibitory interneurons as the point of origin, which are lost early in AD. However, while plaques can associate with neuron cell bodies, evidence increasingly points to the importance of synapses in AD, which localize both to neurites and neuron soma. In fact, the earliest increases in, and aggregation of, Aβ42 in brain have been seen in neurite terminals and dystrophies by immuno-electron microscopy [33, 35]. Distended axonal dystrophies have also been reported to accumulate BACE1 [14] as well as APP [5], and thus, as seen in the outer molecular layer of the dentate (Fig. 7e), such axonal dystrophies can give rise to plaques. However, this does not exclude a role of dendrites as a source of Aβ in plaques in other brain areas, since the loss of markers for dendrites, such as MAP2 [34], occurs early with concomitant Aβ accumulation. Also, dystrophic dendrites distend but not to the extent of large dystrophic axons, making dendritic dystrophies difficult to detect in brain other than by electron microscopy. Since Aβ can target [18] and be internalized at [42] synapses, we envision that injected prion-like Aβ in vivo likely is internalized at synapses. We previously showed in cultured neurons that Aβ42 internalized into neurites can aggregate into fibrils in endosomes and then even extend from intracellular to extracellular at neurite terminals, providing a scenario for extracellular Aβ deposition from prior intracellular aggregation in neurites [42]. Our present data with unilateral hippocampal injection suggest that internalized Aβ in the LEC perforant path first augments Aβ production, increasing production in the soma, and then induces redistribution of Aβ away from the soma to the axon terminals, eventually leading to the induction of extracellular aggregates at LEC axon terminals.

Another mystery in the pathogenesis of AD is the cause of the massive increase of Aβ in brain. In a teenage non-AD brain, the total levels of grey matter Aβ are as low as 1 ng/g compared to upwards of 13,000 ng/g in AD subjects [28], a difference of more than 4 orders of magnitude. In contrast, in a non-AD cohort of adults in their fifties, Aβ levels were only 15–50 times lower than in AD subjects [28]; though, in absolute terms, the amount of Aβ in a healthy middle-aged person is still much closer to levels in a teenager than to that of an AD patient. Thus, there is an increase in brain Aβ with aging and an enormous increase with AD, particularly in the less secreted but more aggregation-prone Aβ42. For comparison, total tau levels are unchanged in AD; though insoluble tau is increased 20-fold, soluble tau is concomitantly decreased [23]. Therefore, in AD, either less Aβ42 is degraded and/or more is produced. In healthy young people, most Aβ is in the EC space comprised of ISF and CSF, which have approximately the same concentrations of Aβ [1], with Aβ42 levels in CSF around 0.7 ng/ml and Aβ40 levels around 5 ng/ml [12]. One can then calculate that in a young healthy brain with approximately 500 ml of grey matter with 140 ml of CSF and 200 ml of ISF, there would be about 2 µg of Aβ in ISF/CSF and 0.5 µg in brain. In AD, there is a characteristic decline of Aβ42 in CSF, but in total, still 1–2 µg of Aβ in ISF/CSF, while in brain, Aβ is increased to about 6000 µg. These numbers are even more dramatic when one considers that most brain Aβ is Aβ42, while in ISF/CSF, Aβ40 predominates. To account for a middle-aged brain going from around 100 µg of brain Aβ42 to the 5000 µg found in an AD brain would require a linear deposition rate of 28 ng Aβ42/h over 20 years. CSF contains 0.7 ng Aβ42/ml and is normally eliminated at a rate of 20 ml/h, so if all CSF Aβ42 was deposited (which it is not) that would account for only half of its deposition in AD. Aβ degradation outside of CSF/ISF also plays a role [36], but increased Aβ production in AD is plausible. We hypothesize that the accumulation of intracellular prion-like Aβ and a disruption of the normal equilibrium between IC and EC Aβ can help explain the massive increase in Aβ that occurs in AD. We show that the normal equilibrium between EC and IC Aβ breaks down with IC accumulation, and in particular, with prion-like accumulation of Aβ. In our model systems, low EC and high IC levels of Aβ both increase Aβ production. Thus, both of the earliest changes in AD, a decline of EC Aβ42 [12] and accumulation of IC Aβ [9], could be due to the formation of intracellular prion-like Aβ, resulting in the increased Aβ production necessary for the massive amount of Aβ in the AD brain. These results have implications for Aβ lowering therapies and may help explain failures of clinical trials targeting Aβ. To be truly effective, treatments might need to effectively target intracellular Aβ. Furthermore, more attention must be paid to how therapies directed against Aβ affect the actively regulated equilibrium between extracellular and intracellular Aβ.

## Conclusions

We show that intracellular prion-like Aβ induces Aβ pathology in vivo in susceptible mice and that injection of AD brain material does not only, as previously described, accelerate extracellular plaque formation, but has effects on intracellular Aβ in anatomically connected areas. Thus, the unilateral injections not only accelerate plaque formation but also intracellular Aβ changes seen in AD, likely, via both redistribution of Aβ within neurons and equilibrium mechanisms mediated by both intracellular and extracellular aggregates of Aβ. To understand the pathogenesis of AD better knowledge of the intracellular changes of Aβ and its extra-intracellular equilibrium is essential.

## Supplementary Information

Below is the link to the electronic supplementary material.

Supplementary file1 Online resource figure legends. Fig. S1, online resource Measuring intracellular Aβ. (a) A fluorescent image of entorhinal cortex (ErC) is split into its NeuN (magenta) and Aβ (green) component. NeuN is then converted to a binary image either positive for NeuN (black) or negative (white). The binary NeuN is subtracted from the Aβ and thus only Aβ signal that overlaps with NeuN will remain. An ROI is then applied over layer II of ErC. This image can then be thresholded on pixel intensity and the analyzed particle command can be used to obtain area of Aβ signal as well as number of puncta; a maximum size is used to exclude plaques. (b) One can also measure the total fluorescence in an ROI of layer II neurons excluding plaques. Here we have quantified ratios of total fluorescence of the injected:uninjected side in the ErC 6 weeks post-injection (one sample t-test p = 0.0387, discrepancy = 0.077, SD of discrepancy = 0.044, df = 3 and n = 4). Both methods yield results in the same direction but there is more noise/background when measuring total fluorescence. Thus, we have used thresholding. Fig. S2, online resource Quantification of plaques after brain or prion-like cell injection. Here we see plaques in the hippocampus of a mouse injected with brain homogenate 16 weeks previously. Plaques are quantified by first using thresholding to define them, then making an ROI based on DAPI, so that plaques are not seen when making the ROI, and finally the analyze particles command is used. Quantifications were done in Fiji 2 and thresholds are always identical when comparing pairs. Fig. S3, online resource In WT mice the injected AD brain material is faint and stays in white matter tracts. (a) WT mouse unilaterally injected with PSEN1 brain homogenate mixed with 0.8 µl india ink and sacrificed 6 weeks post injection. Note the faint Aβ labelling in the external capsule which co-localizes with India ink. (b) For comparison in a 5xFAD mouse unilaterally injected with APP/PSEN1 brain homogenate we also see Aβ labelling in the external capsule but here it is much stronger indicating that even in white matter tracts most of the Aβ signal is from endogenous Aβ. Fig. S4, online resource Strong white matter and cortical Aβ labelling in the injected side. 5xFAD mouse injected with APP/PSEN1 brain homogenate at 7 weeks of age and 10 weeks later the brain was collected. While there is some induced plaque formation in the hippocampus, the clearest differences are in the corpus callosum and the CA1 stratum oriens below, as well as the cortex above. This mouse may have been injected more dorsally than the others. Fig. S5, online resource Posterior parts of mouse brains 4 and 6 weeks post-injection and anterior parts 10 and 16 weeks post-injection. At 4 and 6 weeks no induced Aβ aggregates can be seen in the injected right side. At 10 and 16 weeks induced Aβ aggregates are present even in the anterior parts of hippocampus (arrows) but in contrast to 4 and 6 weeks post-injection induced aggregates are not present in/around the external capsule or corpus callosum. Fig. S6, online resource Full western blots from Figures 5 and 6 and control values from Figure 5. (a): The lanes shown in Fig. 5b and 5e are noted in the red boxes. (b): The lanes shown in 5c are noted with red boxes. Note the large amounts of Aβ (due to treatment with synthetic Aβ) in the 3 middle lanes and the lack of dispersion into neighbouring lanes. Also note that long exposure is required to visualise C99. (c): The lanes shown in Fig. 5d are noted with red boxes. Also note here the large amounts of Aβ in the middle lanes without dispersion into neighbouring lanes. (d): The lanes shown in Fig. 5f are noted with a red box. Primary neurons have lower amounts of Aβ and C99 compared to N2a APPSWE cells and require very long exposure times. (e): The lanes shown in Fig. 5g are noted with a red box. Densitometric quantification of actin for the first two lanes in the red box (DAPT-treated cells) yields a ratio of 43:57 and for the following two (chloroquine-treated cells) it is 59:41. (f): The lanes shown in Fig. 6b are noted with a red box. (g): Fig. 5b with control values shown. (h): Fig. 5c with control values shown. (i): Fig. 5d with control values shown. (j): Fig. 5e with control values shown. Fig. S7, online resource Large amounts of added synthetic Aβ also increases C99. (a) Representative immunoblots in N2a APPSWE cells that have been treated with 1 µM Aβ42 for 6 or 24 hours show increases in both Aβ and C99. (b) Densitometric quantification showing significantly increased levels of C99 at 6 and 24 hours after addition of Aβ (one-way ANOVA Sidak’s multiple comparison test p = 0.0008 and p = 0.0129, df = 12 for both, n = 4 for both, mean difference was 2.40 and 1.63 respectively SE of difference = 0.496). (c) Densitometric quantification showing that Aβ levels are more than twice as high 6 hours after addition of Aβ compared to 24 hours after (two-tailed t-test p = 0.0069, df = 4, mean difference = 43.6, SEM = 8.53 and n = 3). Fig. S8, online resource WT neurons do not develop beaded MAP2 or redistribution of Aβ. (a) 28 DIV WT primary neurons treated with brain ultra- centrifugate from either APP/PSEN1 mouse or WT mouse. In contrast to the transgenic neurons, the WT neurons do not undergo beading of MAP2 or redistribution of Aβ42 from soma to processes even when treated with APP/PSEN1 brain ultra-centrifugate. (b) Fig. 7E but with mCherry in magenta to aid those with difficulty distinguishing red and green. (PDF 11225 kb)
