# Supplementary material for: Neuronal spreading and plaque induction of intracellular Aβ and its disruption of Aβ homeostasis
Source: Acta Neuropathol. 2021 Jul 16;142(4):669–87. doi: 10.1007/s00401-021-02345-9 (PMC8423700; doi:10.1007/s00401-021-02345-9)

Fig S1, online resource

a

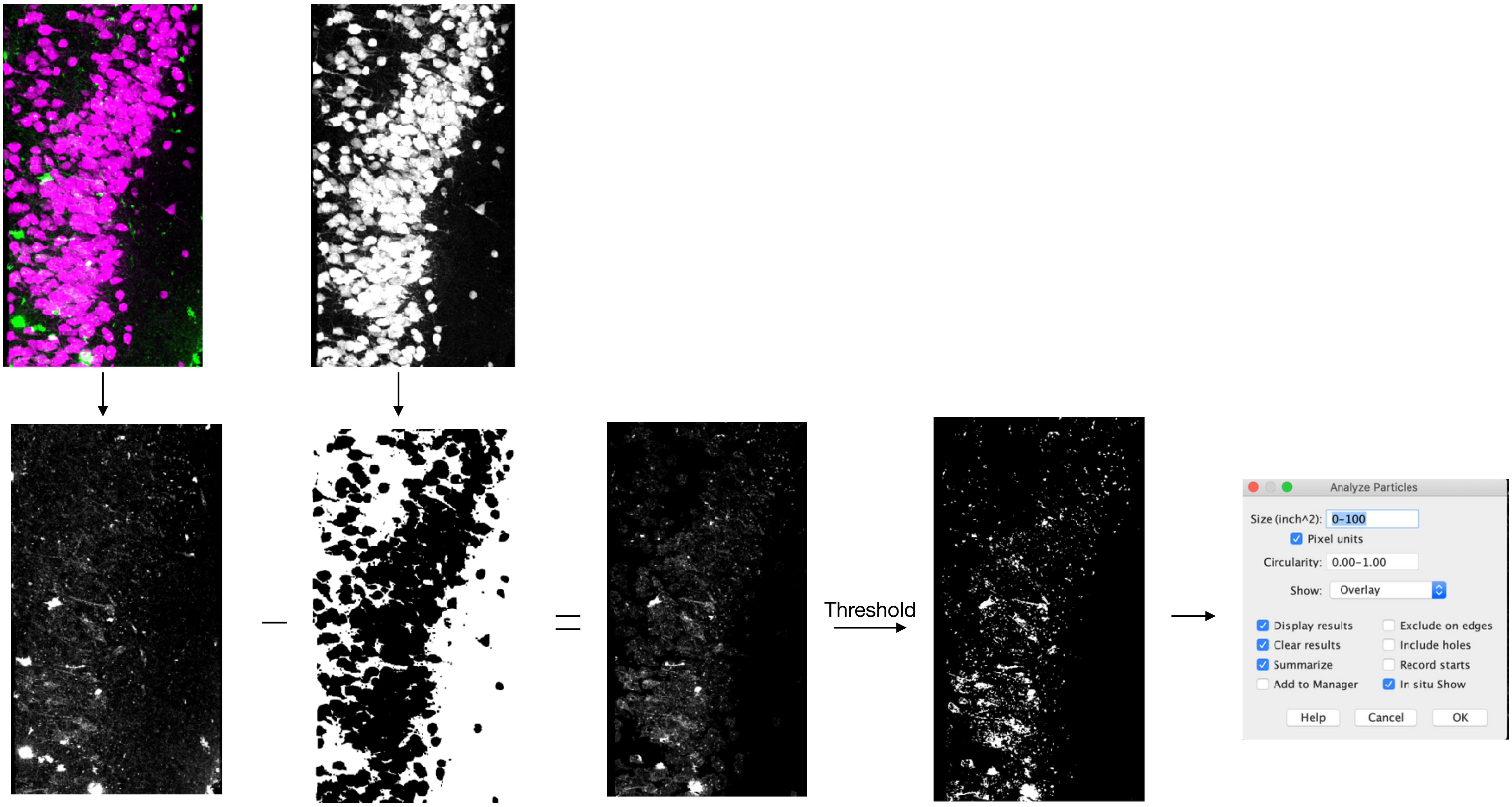

b

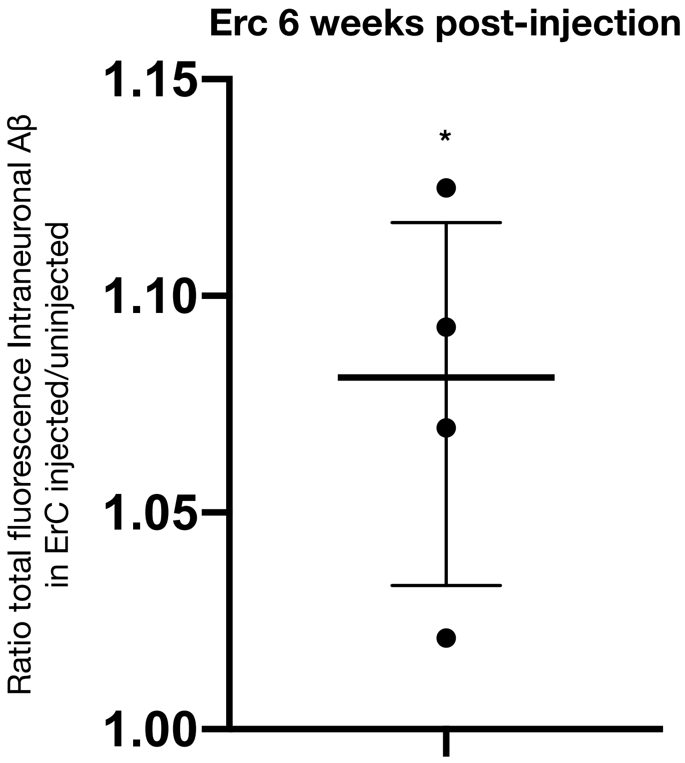

Fig S2, online resource

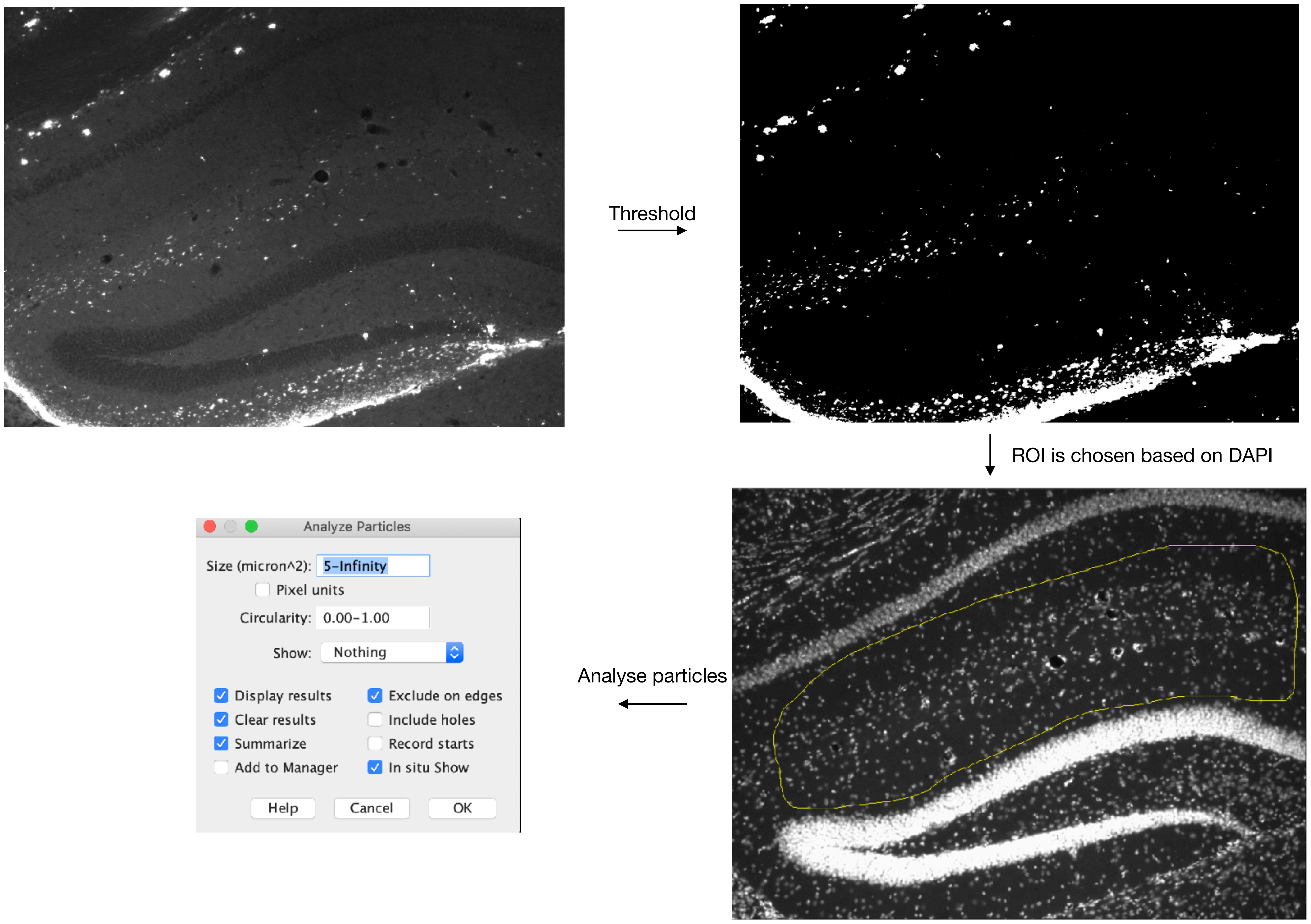

Fig S3, online resource

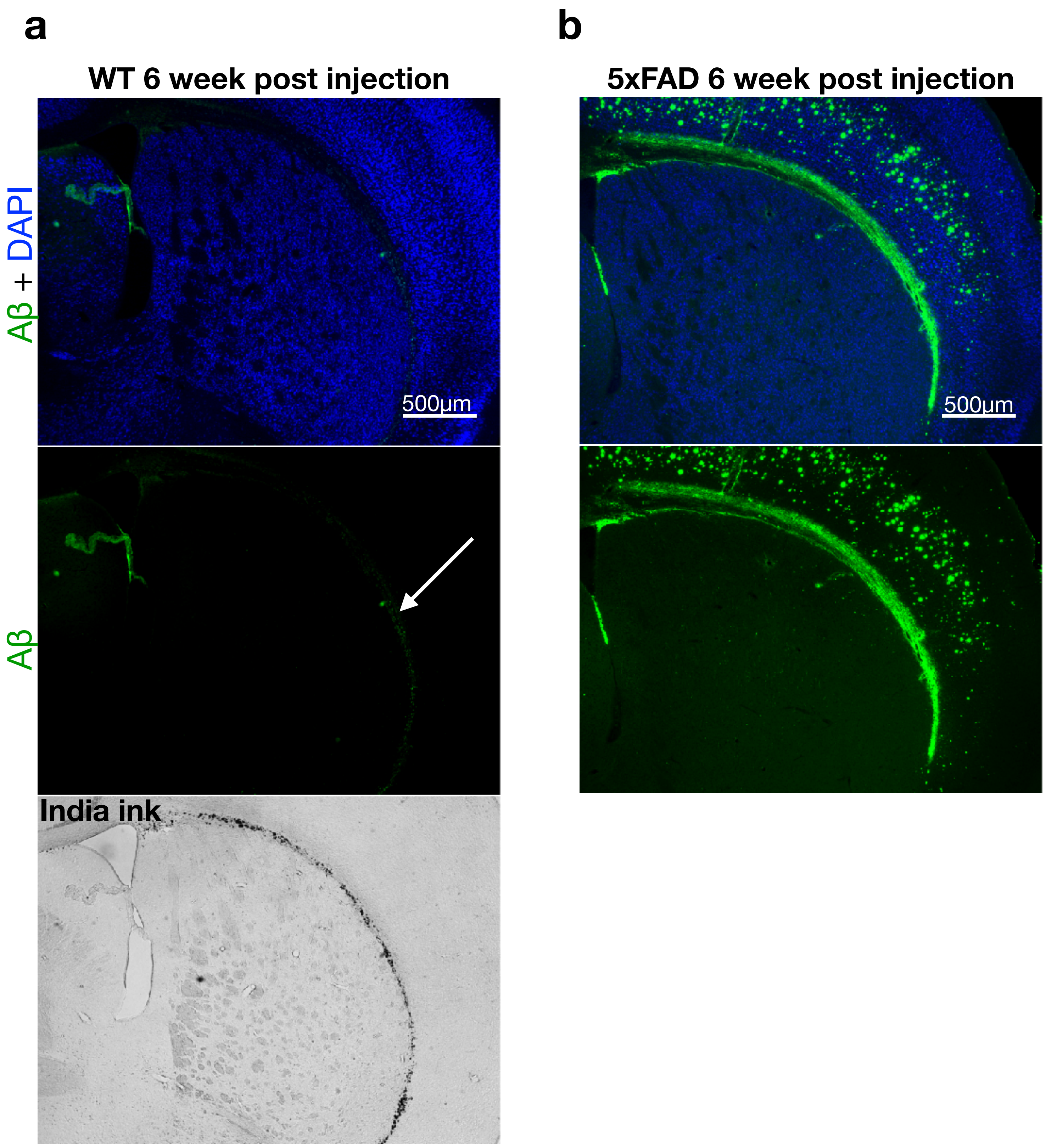

Fig S4, online resource

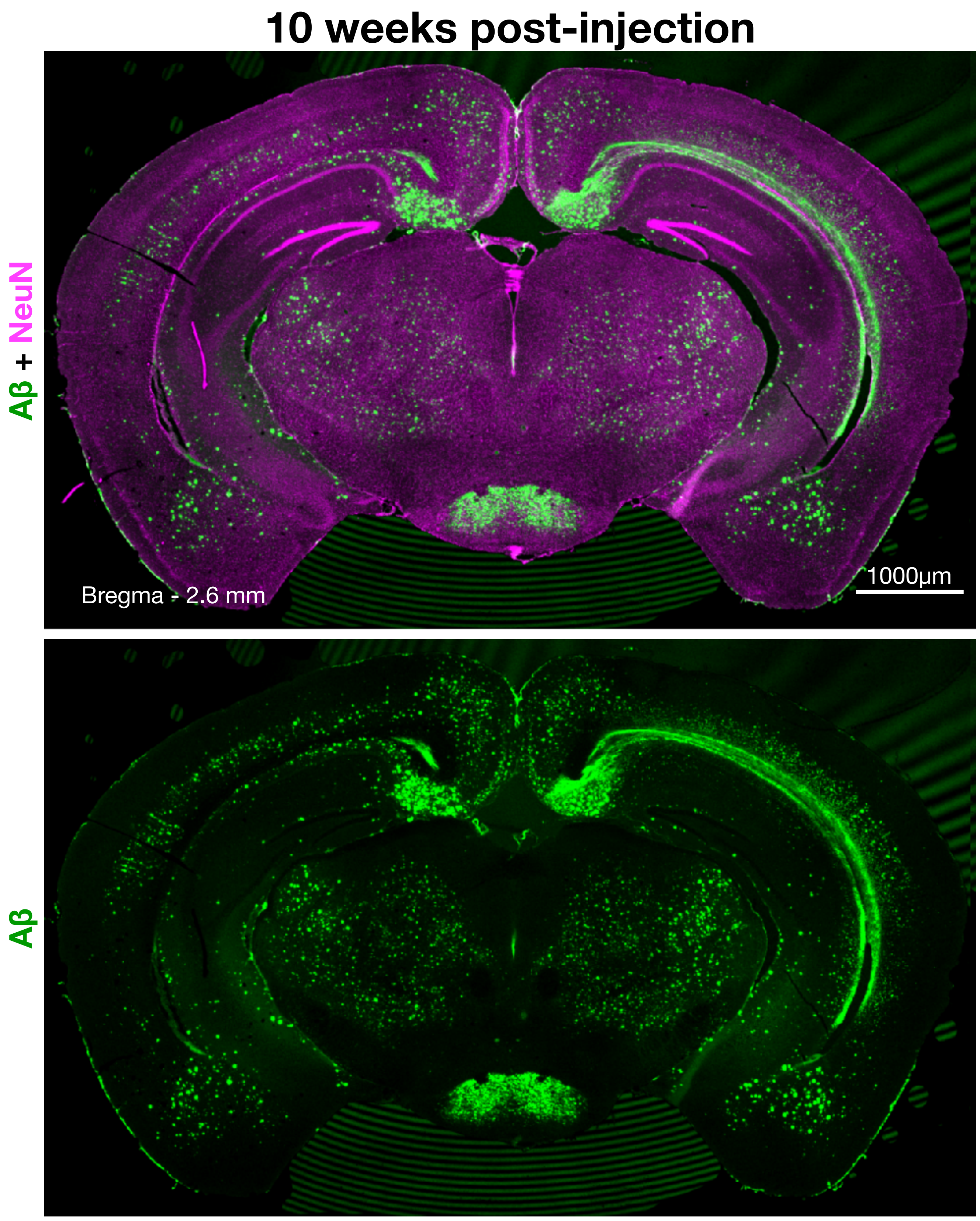

Fig S5, online resource

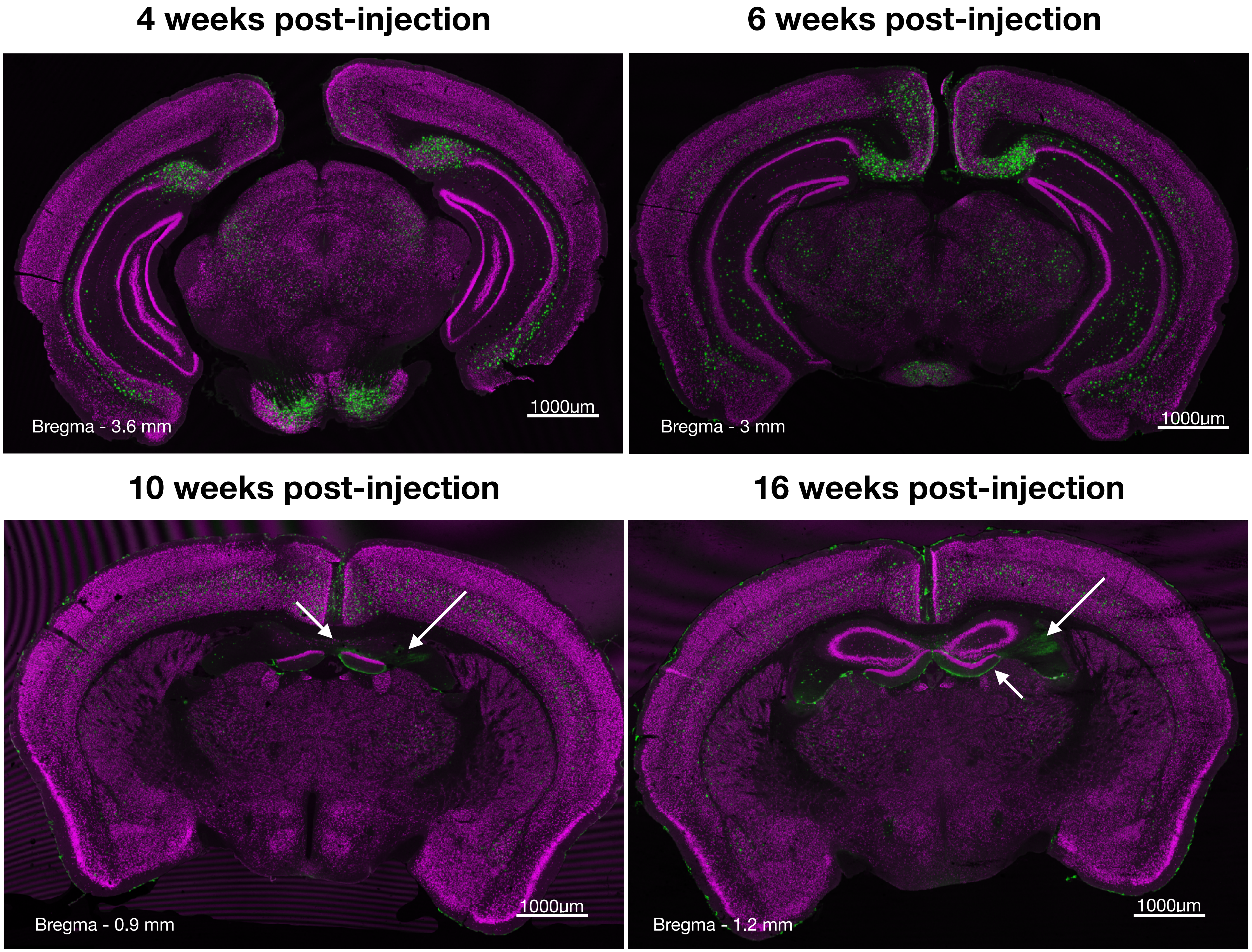

Fig S6, online resource

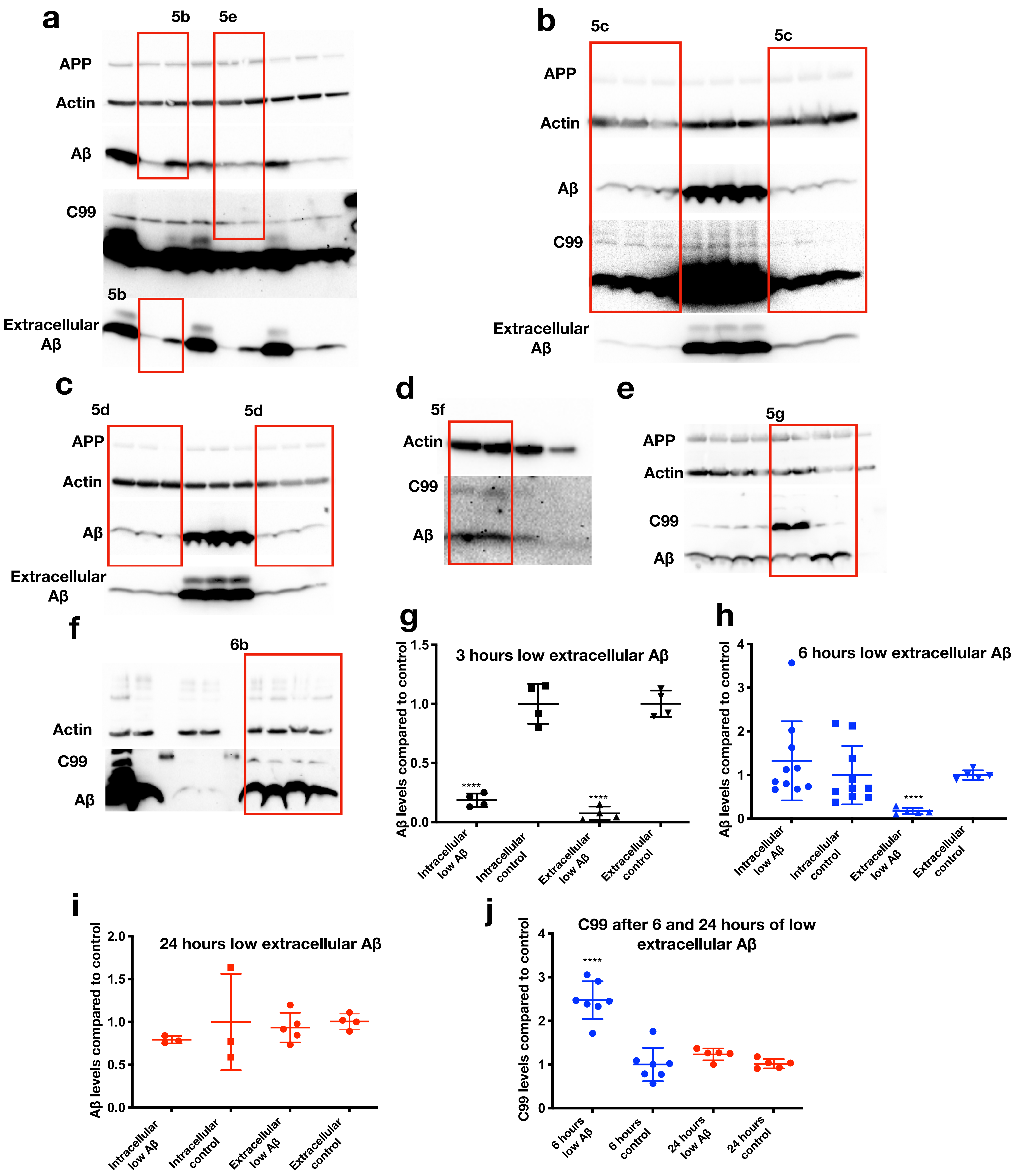

Fig S7, online resource

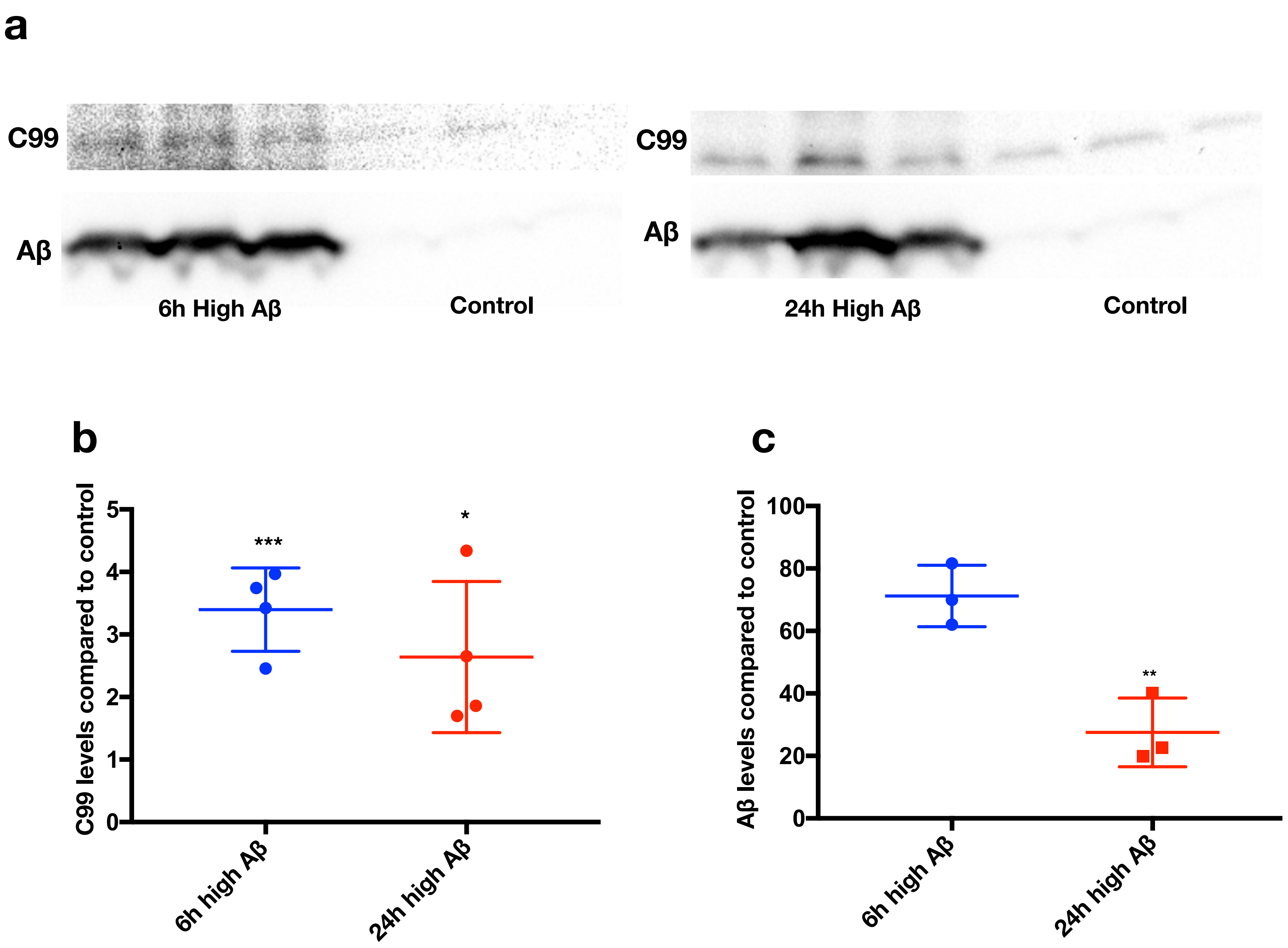

Fig S8, online resource

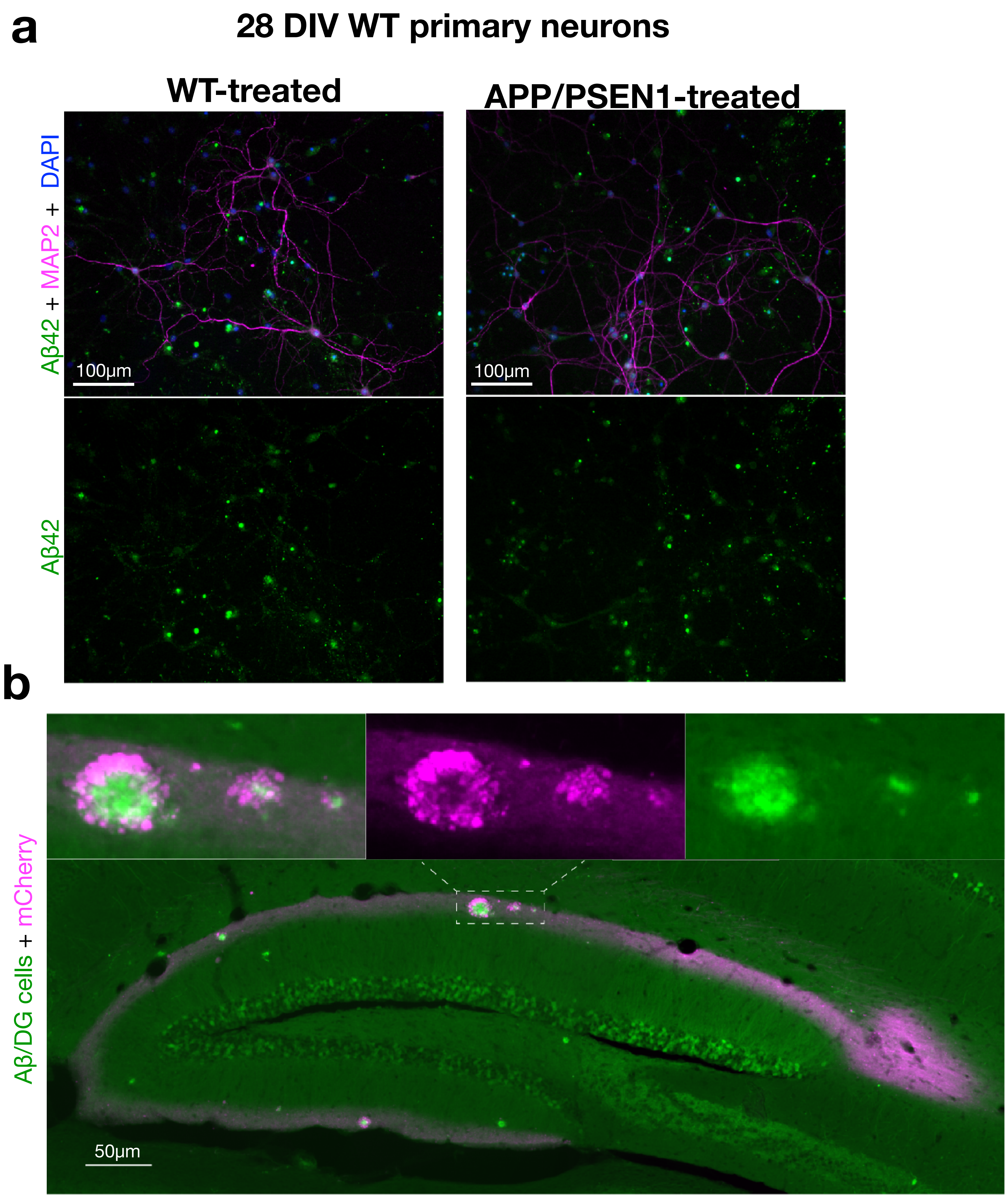

Supplement: Supplementary file 1 — Supplementary file1 Online resource figure legends. Fig. S1, online resource Measuring intracellular Aβ. (a) A fluorescent image of entorhinal cortex (ErC) is split into its NeuN (magenta) and Aβ (green) component. NeuN is then converted to a binary image either positive for NeuN (black) or negative (white). The binary NeuN is subtracted from the Aβ and thus only Aβ signal that overlaps with NeuN will remain. An ROI is then applied over layer II of ErC. This image can then be thresholded on pixel intensity and the analyzed particle command can be used to obtain area of Aβ signal as well as number of puncta; a maximum size is used to exclude plaques. (b) One can also measure the total fluorescence in an ROI of layer II neurons excluding plaques. Here we have quantified ratios of total fluorescence of the injected:uninjected side in the ErC 6 weeks post-injection (one sample t-test p = 0.0387, discrepancy = 0.077, SD of discrepancy = 0.044, df = 3 and n = 4). Both methods yield results in the same direction but there is more noise/background when measuring total fluorescence. Thus, we have used thresholding. Fig. S2, online resource Quantification of plaques after brain or prion-like cell injection. Here we see plaques in the hippocampus of a mouse injected with brain homogenate 16 weeks previously. Plaques are quantified by first using thresholding to define them, then making an ROI based on DAPI, so that plaques are not seen when making the ROI, and finally the analyze particles command is used. Quantifications were done in Fiji 2 and thresholds are always identical when comparing pairs. Fig. S3, online resource In WT mice the injected AD brain material is faint and stays in white matter tracts. (a) WT mouse unilaterally injected with PSEN1 brain homogenate mixed with 0.8 µl india ink and sacrificed 6 weeks post injection. Note the faint Aβ labelling in the external capsule which co-localizes with India ink. (b) For comparison in a 5xFAD mouse unilaterally injected [file 401_2021_2345_MOESM1_ESM.pdf]
